# Unsymmetrical *β*‐Fused Blatter Radical Zinc Phthalocyanines

**DOI:** 10.1002/chem.202503294

**Published:** 2025-12-14

**Authors:** Adrián Hernández, Georgia A. Zissimou, Javier Ortiz, Andreas Kourtellaris, Christos P. Constantinides, Daniel B. Lawson, Ziqi Hu, Eugenio Coronado, Panayiotis A. Koutentis, Ángela Sastre‐Santos

**Affiliations:** ^1^ Área de Química Orgánica Instituto De Bioingeniería, Universidad Miguel Hernández Elche Spain; ^2^ Department of Chemistry University of Cyprus Nicosia Cyprus; ^3^ Department of Life Sciences School of Sciences European University Cyprus, Engomi Nicosia Cyprus; ^4^ Department of Natural Sciences University of Michigan – Dearborn Dearborn Michigan USA; ^5^ Instituto De Ciencia Molecular (ICMol) Universitat De València Paterna Spain

**Keywords:** Blatter‐radicals, phthalocyanine, X‐Ray structure

## Abstract

Blatter‐type radicals defy conventional chemical expectations, combining unusual stability with intriguing electronic and magnetic properties. We report the first phthalocyanines fused at the periphery with a Blatter‐type radical, creating an extended *π*‐conjugated framework. These hybrids merge the characteristic optical and electronic properties of phthalocyanines with a robust, spin‐bearing radical, maintaining remarkable stability under ambient conditions. Spectroscopic, crystallographic, and computational studies reveal their distinctive electronic structure, offering insights into radical *π*‐conjugated systems.

## Graphical abstract

Herein, we report the first synthesis of a new class of β‐fused Blatter–Zn phthalocyanines featuring a persistent radical π‐system, π–π‐driven crystal packing, and SOMO‐centered redox behavior. Single‐crystal X‐ray diffraction shows packing motifs dominated by centrosymmetric *π*‐*π* dimers. DFT reveals cooperative spin communication, establishing a platform for tunable spin and optical materials.

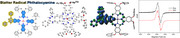

## 1 Introduction

Phthalocyanines are conjugated planar aromatic macrocycles known for their exceptional chemical and thermal stability [1, 2, 3]. Their intense absorption of visible light together with their semiconducting properties make them ideal for applications in organic electronic devices [4, 5] and solar cells [6, 7, 8, 9]. Their ability to form complexes with various metals and ligand expands their interest in the design and development of materials with specific properties, such as gas sensors [10], photocatalysis [11], metal‐organic frameworks (MOFs) [12, 13, 14] and magnetic materials [15, 16]. Moreover, their ability to interact with biomolecules and their low toxicity make them promising candidates in photodynamic therapies for the treatment of diseases [17, 18, 19].

The 1,2,4‐benzotriazin‐4‐yl radical, or “Blatter's radical,” was first reported in 1968 [20]. It is thermodynamically stable and tolerates both oxygen and moisture under ambient conditions, owing to extensive delocalization of the unpaired electron over the three nitrogen atoms and the fused benzene ring [21, 22]. Blatter‐type radicals exhibit remarkable properties, including ferromagnetic [23, 24, 25, 26, 27, 28, 29, 30, 31, 32] and antiferromagnetic [23, 24, 25, 26, 27, 28, 29] interactions, a narrow electrochemical window [33, 34, 35], and low excitation energies [36, 37, 38]. They have found applications as initiators for controlled polymerizations [39, 40, 41, 42], as components of metal coordination complexes [43, 44, 45, 46, 47, 48, 49], and in MOFs [50], as well as in molecular electronics [51], photodetectors [52], liquid crystal photoconductors [53], and electroactive polymers for purely organic batteries [41, 54, 55, 56].

Recent studies further highlight the versatility of Blatter radicals. Their structural robustness is evidenced by investigations of polymorphism and high‐pressure phase transitions [57, 58]. Applications have expanded to include symmetrical redox‐flow batteries [59].

In spintronic contexts, Blatter radicals exhibit reversible Kondo‐state switching on metal surfaces [60], and at transition‐metal interfaces such as polycrystalline cobalt [61]. They also retain open‐shell character in gold–molecule–gold junctions [62, 63, 64, 65]. They can form optically distinguishable spin‐isomers [66], and high‐spin radicals with robust stability, electrical conductivity, and efficient photothermal conversion [67, 68, 69, 70, 71, 72], as well as electronically perturbed vibrational excitations in luminescent Blatter radicals [73].

Complementary computational studies on methyl‐driven Overhauser dynamic nuclear polarization (DNP) agents [74, 75, 76] and investigations of metal‐free organic radical spin sources [77, 78] illustrate the tunability of their electronic and spin properties, highlighting their potential as multifunctional materials for molecular electronics, spintronics, and energy applications [79, 80].

Recently, 1,3‐diphenyl‐1,4‐dihydrobenzo[*e*][1,2,4]triazin‐4‐yl‐6,7‐dicarbonitrile has been shown to be a remarkably stable, electron‐deficient Blatter radical with intriguing solid‐state magnetic properties [29]. The two nitrile groups enhance radical delocalization and crystallinity, enabling its use as a phthalonitrile in cross‐condensation reactions to produce stable, radical‐containing asymmetric phthalocyanines. These compounds combine the characteristic properties of phthalocyanines with the additional functionality of an unpaired electron, making them promising candidates for electronic and spintronic applications.

While spin‐active metal centers in phthalocyanines are well‐known (*e.g*., Co^II^ or Mn^II^) [81, 82], covalent functionalization of the macrocycle with stable organic radicals is less common. Previous efforts have focused primarily on *σ*‐bound nitroxide radical substituents appended peripherally [83, 84, 85] or coordinated axially to the central metal [86, 87]. These include widely studied 2,2,6,6‐tetramethylpiperidine‐1‐oxyl (TEMPO) derivatives, which have been used to probe intermolecular magnetic interactions and modify physicochemical properties of Pcs [83, 84, 86, 87]. Recently, unstable *π* radicals were formed via pyrrolic H atom abstraction from H_2_Pc on a MoS_2_ decoupling layer, demonstrating current interest in macrocycle‐centered spin [88]. (Note: ESR signals can also originate from charge‐transfer interactions between diamagnetic Pc cations and dioxygen anions stabilized on a host surface [89]).

Our work, in contrast, introduces a highly stable, electron‐deficient Blatter radical that is fused into the Pc *β*‐position using the 6,7‐dicarbonitrile building block. This strategy enables the direct integration of a robust, spin‐bearing unit into the macrocycle's *π* system via cross‐condensation, leading to a new class of asymmetric Pc hybrids. The distinct molecular architecture, created by directly integrating an open‐shell Blatter radical with a closed‐shell phthalocyanine π‐system, not only enables finely tunable spin and optical properties but also provides an essential foundation for designing next‐generation functional materials [79]. Owing to its intentional structural coupling of radical and chromophore units, this system emerges as a promising powerful model for probing spin–photon interactions in radical–chromophore hybrids, an area of growing interest for molecular spintronics and quantum information science [60, 77, 78, 90].

**CHART 1:**
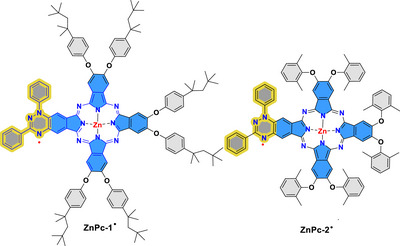
Structure of ZnPc‐1· and ZnPc‐2·.

In this paper, we describe the first synthesis of asymmetric zinc phthalocyanines with a Blatter radical as a substituent. Two derivatives have been prepared: **ZnPc‐1**, with 4‐*tert*‐octylphenoxyl substituents to increase its solubility, and **ZnPc‐2**, with bis(1,6‐dimethylphenoxyl) groups to boost its crystallinity, allowing us to get single‐crystal X‐ray structures. Chart 1

### 1.1 Synthesis and Characterization

**SCHEME 1:**
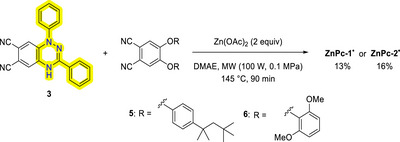
Synthetic route of **ZnPc‐1** and **ZnPc‐2·**.

The synthesis of asymmetrical metal phthalocyanines involved a statistical cyclotetramerization reaction between phthalonitrile **3**, the respective phthalonitrile (**5** or **6**), and zinc acetate (see Supporting Information). Phthalonitriles **3–4** [29], **5** [91] and **6** [92] were synthesized in accordance with established literature procedures Scheme 1.

Following purification via column chromatography, **ZnPc‐1·** and **ZnPc‐2·** were obtained as green powders with yields of 13% and 16%, respectively. Their ^1^H NMR spectra showed a very broad band in the aromatic zone corresponding to the phenolic substituents and no signal for the hydrogens on the phthalocyanine ring. When these samples were treated with ascorbic acid, an instantaneous change from green to blue color was observed. Now, the reduced **ZnPc‐1H** spectrum exhibited sharp aromatic and aliphatic signals, with the isoindole unit and aromatic substituent peaks between 7.20 and 9.01 ppm and alkyl chain peaks within 0.83 and 1.88 ppm. The spectrum of reduced **ZnPc‐2H** revealed signals from the core of the phthalocyanine and aromatic substituents (7.34 to 8.60 ppm), along with a peak corresponding to methyl groups at 2.42 ppm (see SI). From these observations we deduced that the initially isolated green products were the radical species. When the phthalocyanine synthesis reactions were repeated with radical phthalonitrile **4**, the same results were obtained.

UV−vis spectra were recorded in DMF for both reduced and radical MPcs, displaying the characteristic Soret and Q bands around 350 nm and 600–780 nm, respectively (Table S1). The addition of ascorbic acid to reduce the phthalocyanines resulted in a noticeable change in the UV−vis spectra, characterized by an increased molar extinction coefficient and little blue‐shifted peaks (SI, Figures S4 & S11). The mass spectra of both compounds (HR‐MALDI‐TOF) were also performed in their radical and reduced forms, obtaining in both cases the mass of the radical form (SI, Figures S8 & S15). Electrochemical measurements in anhydrous DMF (Fc/Fc^+^ reference) show that both **ZnPc‐1·** and **ZnPc‐2·** exhibit two quasi‐reversible oxidation and two quasi‐reversible reduction processes (SI, Figures S6, 7 & S13, 14). Differential pulse voltammetry (DPV) provided better‐resolved features and was used to determine the formal potentials. **ZnPc‐1·** displays redox events at *E*_Red2_ = −1.48 V, *E*_Red1_ = −1.03 V, *E*_Ox1_ = −0.13 V, and *E*_Ox2_ = 0.48 V, while **ZnPc‐2·** exhibits a similar profile (*E*_Red1_ = −1.05 V, *E*_Ox1_ = −0.12 V, and *E*_Ox2_ = 0.45 V), except for a substantially more negative second reduction at *E*_Red2_ = −1.79 V (SI, Table S1). Estimated SOMO energies derived from *E*_Ox1_ are –4.67 eV (**ZnPc‐1·**) and –4.68 eV (**ZnPc‐2·**), with corresponding LUMO levels of –3.77 and –3.75 eV (see SI for more details, Table S1).

### 1.2 Magnetic Properties

**FIGURE 1:**
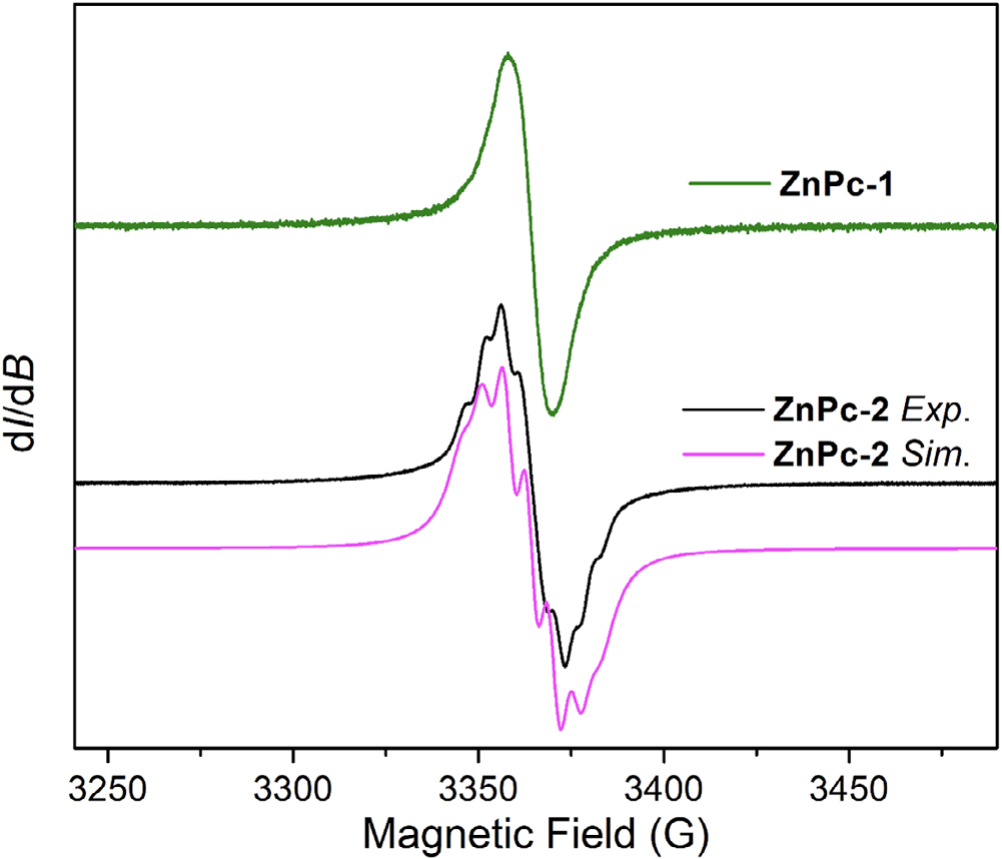
EPR spectra of **ZnPc‐1·** and **ZnPc‐2·** in CH_2_Cl_2_ solutions recorded at room temperature. The simulated EPR spectrum of **ZnPc‐2·** (pink line) is obtained by using an isotropic *g* tensor of 2.0056 and an equal hyperfine coupling constant *A* of 16.5 MHz for the three adjacent N atoms.

The radical nature of **ZnPc‐1·** and **ZnPc‐2·** has been confirmed by X‐band electron paramagnetic spectroscopy (EPR) in CH_2_Cl_2_ solutions at room temperature (Figure 1). While the recorded spectrum of **ZnPc‐1·** shows a single peak with an isotropic *g* tensor of 2.0056, **ZnPc‐2·** features additional splitting ascribed to the hyperfine coupling between the unpaired electron and the *I* = 1 nuclear spin of the three surrounding ^14^N atoms, which thus leads to a hyperfine structure with seven peaks. To fit the experimental EPR spectrum of **ZnPc‐2·**, the spin Hamiltonian can be described as follows:

$$\;{\hat{H}}_{\mathrm{spin}}=g\mu_{B}B\hat{S}+A\hat{S}\hat{I}$$

where $\mu_{B}$ is the Bohr magneton and A denotes the isotropic hyperfine coupling constant. This gives rise to an isotropic *g* tensor of 2.0056 and an equal A value of 16.5 MHz for the three N atoms, indicating that the unpaired electron is delocalized over the triazine ring instead of being localized on one N atom. By contrast, the lack of hyperfine structures for **ZnPc‐1·** suggests a faster spin‐lattice relaxation rate, which is in line with the fact that **ZnPc‐1·** has many more H atoms than **ZnPc‐2·**.

### 1.3 Single‐Crystal X‐ray Diffraction Studies

Single crystals of **ZnPc‐2A** and **B** suitable for X‐ray analysis were grown by vapor diffusion of *n*‐hexane and cyclohexane, respectively, into a 1,2‐dibromoethane (DBE) solution of the complex. Diffraction data were collected at 100(2) K and refined to *R*_1_ ≈ 0.11 with >99% completeness.

**ZnPc‐2A** crystallizes in the monoclinic space group *P*2_1_/*c*, with one radical molecule and three DBE solvent molecules in the asymmetric unit (*V* = 9898.1(3) Å^3^, *Z* = 4) (SI, Tables S2 & S3), while **ZnPc‐2B** also adopts a monoclinic lattice, in the space group *P*2_1_/*n*, with one radical molecule, two cyclohexanes, and one DBE molecule in the asymmetric unit (*V* = 10419.3(3) Å^3^, *Z* = 4) (SI, Tables S4 & S5).

#### 1.3.1 Molecular Structure

**FIGURE 2:**
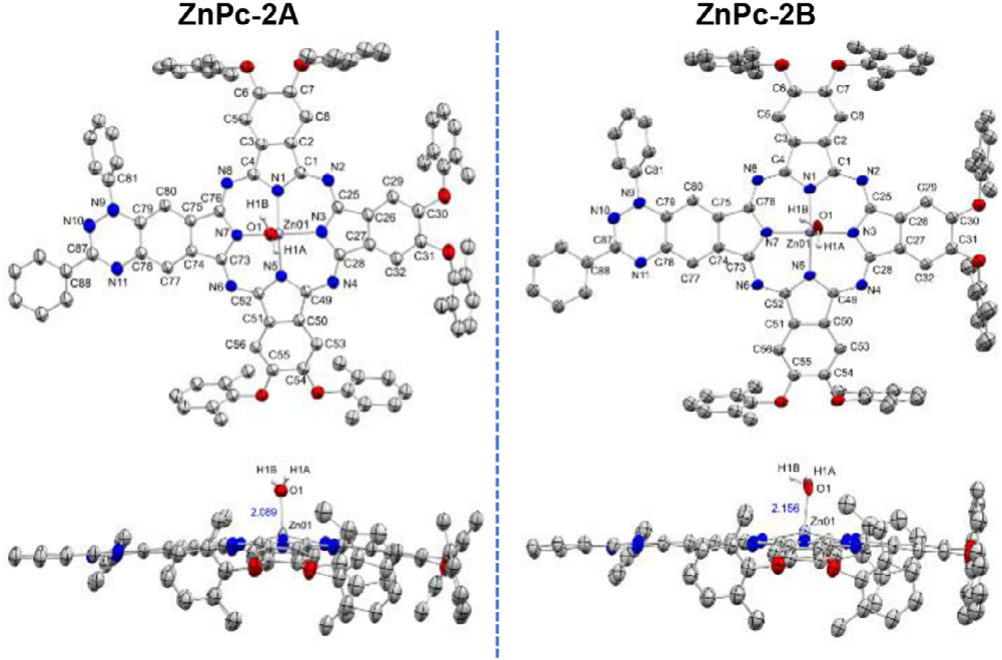
ORTEP representations of **ZnPc‐2·** radicals **A** (left) and **B** (right) in aerial (above‐plane) and side (in‐plane) views. Thermal ellipsoids are drawn at the 50% probability level. Hydrogen atoms (except those of the coordinated water ligand) and cocrystallized solvent molecules are omitted for clarity. Selected atom numbering and the Zn‐O (OH_2_) bond length (Å) are indicated.

In both structures, the phthalocyanine (Pc) macrocycle hosts a central zinc atom axially coordinated by a water molecule [Zn–O1 = 2.089(5) Å (**A**) and 2.156(5) Å (**B**)] (Figure 2 and SI Figures S16 & S17), consistent with a weak Zn–OH_2_ interaction, typically ∼ 2.0‐2.2 Å [93, 94, 95].

The macrocycles adopt a distorted bowl‐shaped conformation, with all four isoindole units bent in the same direction relative to the mean plane of the inner 16‐atom ring. This doming is asymmetric. Quantitative analysis of the benzo ring centroids (SI, Table S6) shows that [1,2,4]triazino[5,6‐*f*]isoindole (TAI) unit is the least displaced from the plane, with centroid deviations of 0.178 Å (**A**) and 0.068 Å (**B**), and maximum atomic deviation of C79: 0.255 Å (**A**) and 0.139 Å (**B**). In each structure, an isoindole unit adjacent to the triazinyl‐fused ring is the most displaced from the macrocyclic plane [C54 in **ZnPc‐2A**: 1.016 Å, centroid: 0.619 Å; C6 in **ZnPc‐2B**: 0.811 Å, centroid 0.516 Å], while the remaining non‐triazinyl units exhibit intermediate deviations, confirming the asymmetric doming. Tentatively, this structural asymmetry is attributed to the rigidity imparted by the fused benzotriazinyl system, which restricts the conformational flexibility of that portion of the macrocycle.

Distortion‐mode decomposition using *PorphyStruct* [96] (SI, Figure S18) supports this interpretation. While centroid analysis shows slightly larger local out‐of‐plane deviations in **ZnPc‐2A**, PorphyStruct reveals that **ZnPc‐2B** exhibits a marginally larger total out‐of‐plane distortion (*D*_oop_ = 0.539 Å vs. 0.495 Å for **A**) due to an enhanced saddling component. Doming remains the dominant deformation mode in both complexes.

The zinc atom resides approximately 0.51 Å out of the plane, consistent with a distorted square‐pyramidal coordination geometry imposed by the axial aqua ligand. Zn–N bond distances (∼2.02‐2.05 Å) and *cis/trans* N–Zn–N angles (∼87° and ∼155°, respectively) are typical of zinc phthalocyanines [93, 94, 95], while phthalocyanine C–N (1.31–1.38 Å) and pyrrole C–C (1.38–1.47 Å) distances fall within the expected ranges for aromatic delocalization (SI, Tables S3 & S5) [97, 98].

The TAI unit, hosting the benzotriazinyl radical, is essentially planar, with minimal deviations within the 13‐atom framework [0.027 Å at C80 (**A**); 0.041 Å at C73 (**B**); SI, Table S7]. The C–C (≈ 1.38–1.46 Å) and C–N (≈ 1.33–1.40 Å) bond lengths (SI, Tables S2 & S4) lie between typical single‐ and double‐bond values, indicative of partial electron delocalization and *π‐*conjugation across the system. The radical core adopts trigonal‐planar geometry (∠C79–N9–N10 ≈ 123°, ∠C87–N10–N9 ≈ 117°, ∠N11–C87–N10 ≈ 126°) (SI, Tables S3 & S5). The N9–phenyl group is twisted out of plane (torsion angles *Φ* ≈ ‐59 to ‐50°), while the C87–phenyl group is nearly coplanar (*Φ* ≈ ‐9.7 to 1.6°), occupying a nodal plane of the radical SOMO and contributing little to conjugation (SI, Table S7). These metrics are consistent with established Blatter radical metrics [99–104] and support a delocalized radical core conjugated with the phthalocyanine.

#### 1.3.2 Solid‐State Packing

**FIGURE 3:**
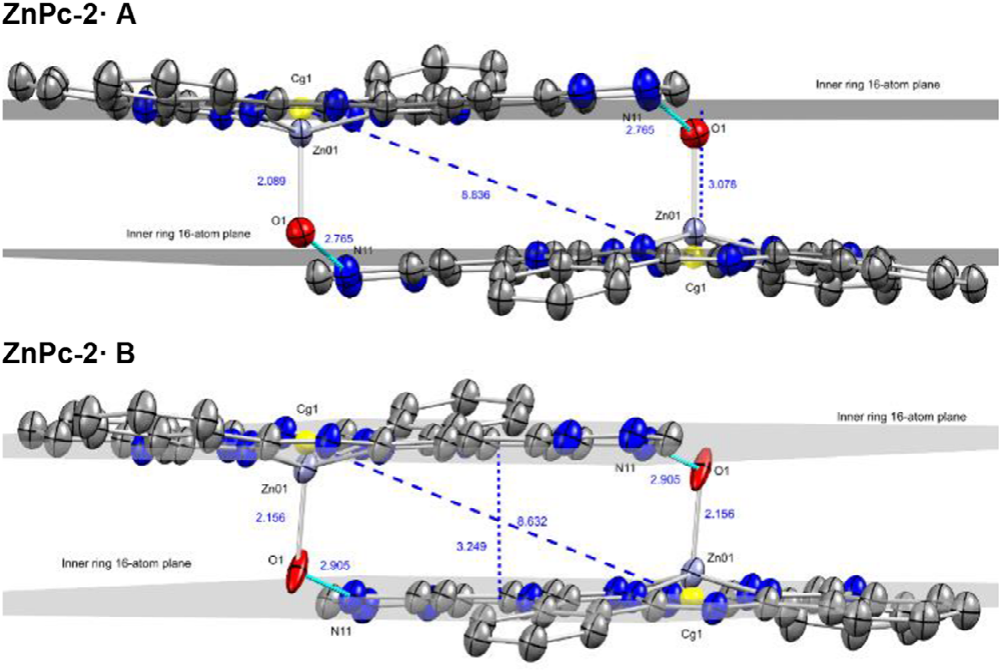
Inversion‐symmetric bowl‐to‐bowl dimers of **ZnPc‐2·** radicals **A** (top) and **B** (bottom), showing selected intradimer interactions and distances (blue, Å). ORTEP views with thermal ellipsoids drawn at the 50% probability level. Solvent molecules, hydrogen atoms, and peripheral bis(1,6‐dimethylphenoxy) and phenyl substituents are omitted for clarity. Phthalocyanine inner ring 16‐atom planes are shown in dark grey, and 16‐atom phthalocyanine centroids (Cg^1^) are shown in yellow.

In the solid state, **ZnPc‐2·** molecules assemble into inversion‐symmetric bowl‐to‐bowl dimers, stabilized by a combination of O–H···N hydrogen bonding and slipped *π–π* stacking between the phthalocyanine cores (Figure 3). The hydrogen bond forms between the Zn‐bound aqua ligand of one molecule and the spin‐bearing triazinyl nitrogen atom (N11) of a symmetry‐related molecule (O1···N11 ≈ 2.8‐2.9 Å [2.765 Å (**A**) and 2.905 Å (**B**)], O1–H1A···N11 = 1.873 Å (A) and 2.060 Å (B), ∠O–H···N ≈ 149–155° [148.95° (**A**) and 155.17° (**B**)], directly linking the metal center to the peripheral radical locus. The *π–π* interaction is defined by the centroids of the inner 16‐atom macrocyclic rings, with separation of 8.6‐8.8 Å [8.836 Å (**A**) and 8.632 Å (**B**)], mean interplanar distances of 3.1‐3.2 Å [3.078 Å (**A**) and 3.249 Å (**B**)], and slippage angles of ∼68‐70° [69.6° (**A**) and 67.9° (**B**)], consistent with the geometry expected for curved *π* systems optimizing their interactions [105]. Together, these interactions form a robust supramolecular bridge within the dimer.

**FIGURE 4:**
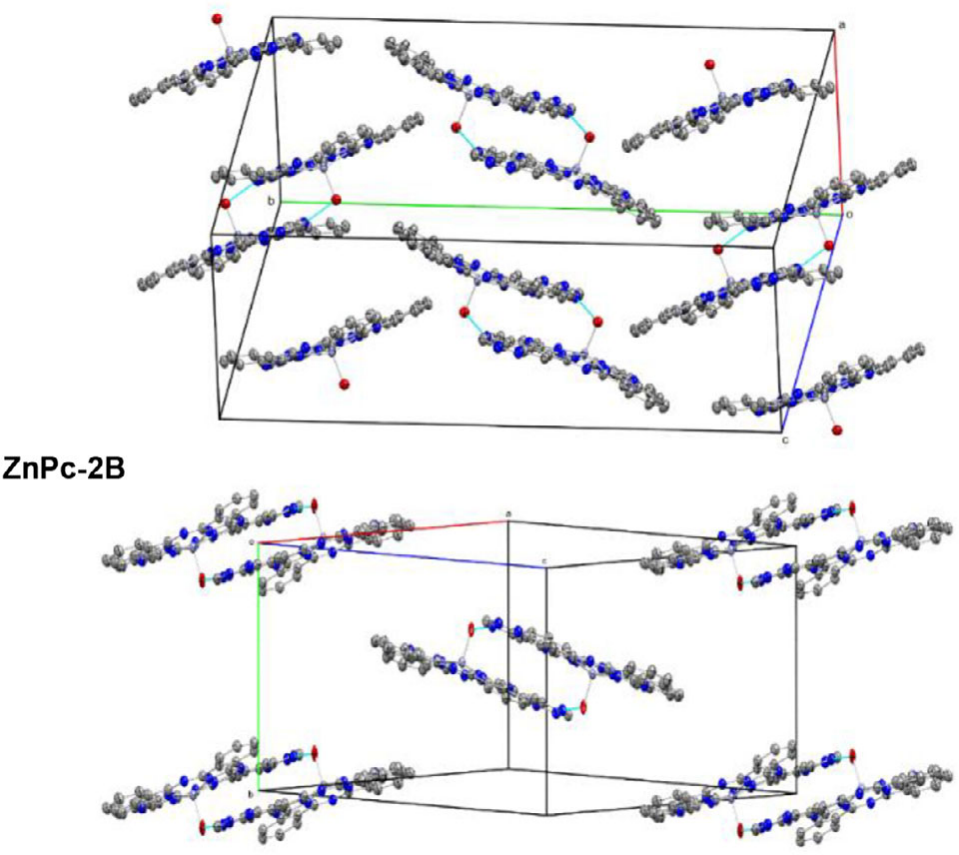
Packing herringbone motifs of **ZnPc‐2·** radicals **A** (above) and **B** (below). ORTEP views with thermal ellipsoids drawn at the 50% probability level. Hydrogen atoms, solvent molecules, and peripheral bis(1,6‐dimethylphenoxy) and phenyl substituents are omitted for clarity. The unit cell axes and O–H···N hydrogen‐bonding interactions are shown.

These dimers further organize into columnar stacks along the *a*‐axis (**A**) or *b*‐axis (**B**), forming herringbone motifs (Figure 4) with angles of 39.98° (**A**) and 41.68° (**B**) (SI, Figure S19). While the molecular conformation is largely unaffected by the solvent, the interstitial cocrystallized solvent molecules (three DBE molecules in **A**; two cyclohexanes plus one DBE in **B**) modulate packing within the columns (see SI, Figure S20 for a view of the 2×2×2 unit cell expansion including the solvent molecules). This results in shorter interdimer separations in **ZnPc‐2A** [7.375 Å (**A**) vs 11.398 Å (**B**); interdimer distance between neighboring phthalocyanine 16‐atom inner ring planes], reflecting a more compact arrangement.

This herringbone motif is further stabilized by a network of weaker C–H···*π* and C–H···O interactions involving the peripheral substituents and cocrystallized solvent molecules, which occupy the interstitial voids (see SI for further discussion, Tables S8 & S9).

Overall, the supramolecular architecture positions the benzotriazinyl radical cores on opposite faces of the inversion‐related dimer, with centroid separations of ≈7.8–8.1 Å for the benzotriazinyl units [7.798 Å (**A**) vs 8.142 Å (**B**)], which is expected to minimize direct through‐space spin–spin interactions. This analysis illustrates how molecular conformation and supramolecular packing, modulated by the crystallizing solvent, dictate the solid‐state organization of these radical‐phthalocyanine hybrids.

### 1.4 Computational Studies

Single‐point (SP) and time‐dependent (TD) DFT calculations were carried out on the X‐ray crystal structures of both **ZnPc‐2· A** and **B** radicals to examine their electronic structures and optical properties (Figure S21). Comparison of the two structures revealed only minor differences in ground‐state energies (Δ*E* = 0.236 Hartree) and frontier molecular orbitals (Δ*E* = 0.006–0.04 eV), primarily due to slight variations in the axial Zn–OH_2_ coordination. Given these small differences, **ZnPc‐2· B**, which is slightly lower in energy (*E*_GS_) of −6495.823 Hartree, and a dipole moment of 3.646 D (Figure S22), is used as the representative system in the discussion (see Supporting Information for comparative data on **ZnPc‐2A**).

**FIGURE 5:**
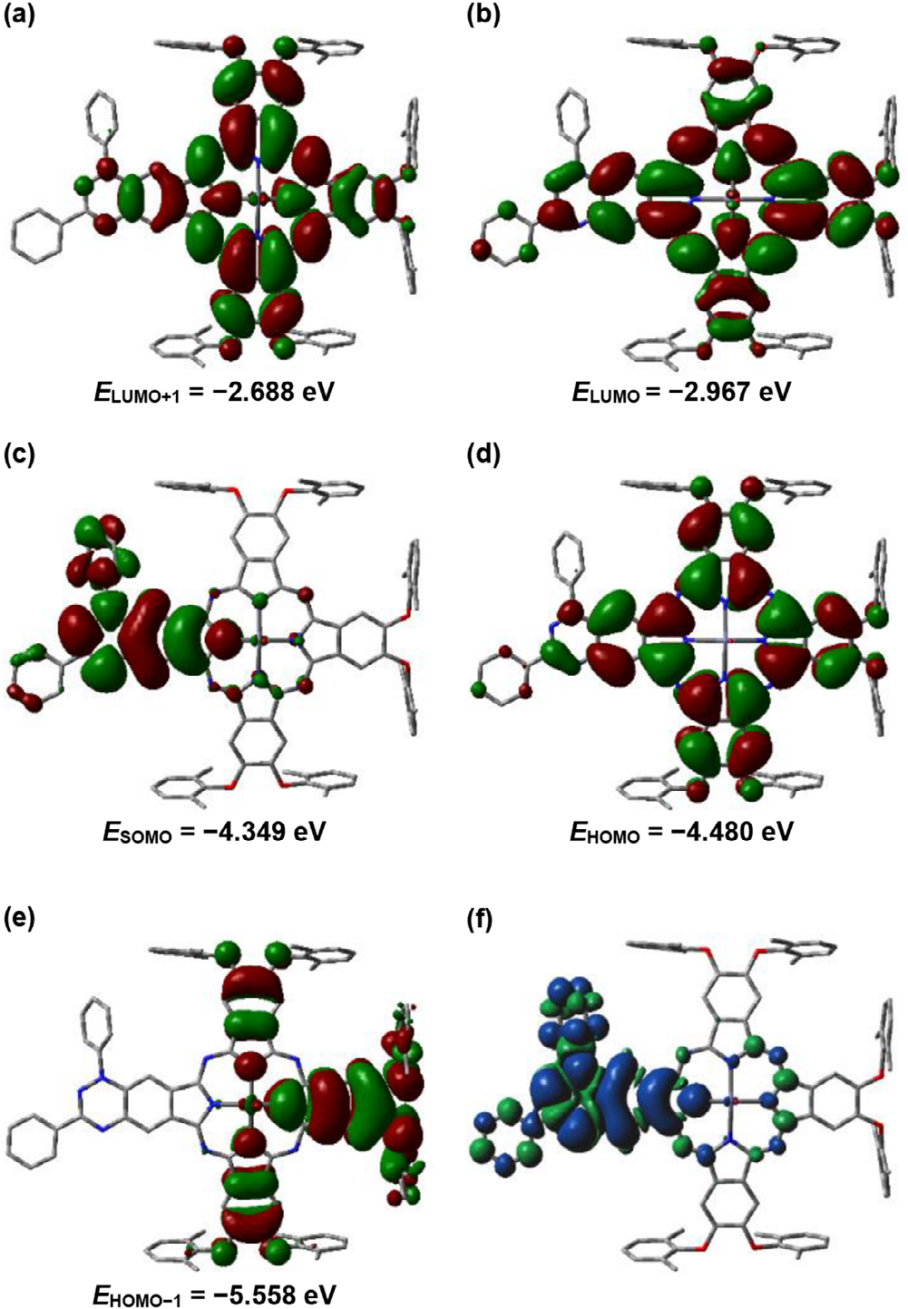
(a)‐(e) Frontier molecular orbital (FMOs) surfaces for **ZnPc‐2B** radical, with the H_2_O ligand, as calculated at the DFT UB3LYP/6‐31G(2d,p) level of theory; (f) Spin density map of **ZnPc‐2·B** with the H_2_O ligand, as calculated with UB3LYP/6‐31G(2d,p). The *α* and *β* spin densities are rendered in blue and green, respectively, using an MO and density isovalues of 0.0002 a.u. The visualization highlights the spatial distribution of unpaired electrons within the molecular framework, emphasizing regions of net spin polarization, as well as the minimal effect of the ligand on the spin distribution.

Analysis of the Frontier Molecular Orbitals (FMOs) reveals a distinct spatial segregation between the radical and macrocycle components, with a SOMO*α*‐LUMO*α* (FMO) energy gap of 1.382 eV (Figures 5a–e & S23). The SOMO*α* is localized predominantly on the TAI radical fragment. In contrast, the HOMO*α*, LUMO*α*, and LUMO+1*α* are delocalized across the phthalocyanine *π* system including, the benzotriazinyl moiety, while the HOMO−1*α* is confined to the core of the macrocycle with minimal contribution from the benzotriazinyl unit. This spatial separation between the SOMO and the other FMOs indicates limited orbital mixing between the radical and the macrocyclic scaffold, consistent with electronic decoupling of the two *π* systems. This arrangement establishes the phthalocyanine HOMO as a redox reservoir, while its LUMO and LUMO+1 orbitals can accept electron density without perturbing the radical center.

The spin density distribution (Figures 5f & S24) corroborates this picture, with the unpaired electron localized mainly on the TAI fragment, in line with the SOMO*α* localization. Minor spin delocalization is observed over the N–C ligand framework coordinating the zinc center, indicating weak but nonnegligible spin communication between the TAI radical and the Zn coordination sphere. Such interactions may enable electronic coupling across the complex and influence its redox or magnetic exchange behavior.

**FIGURE 6:**
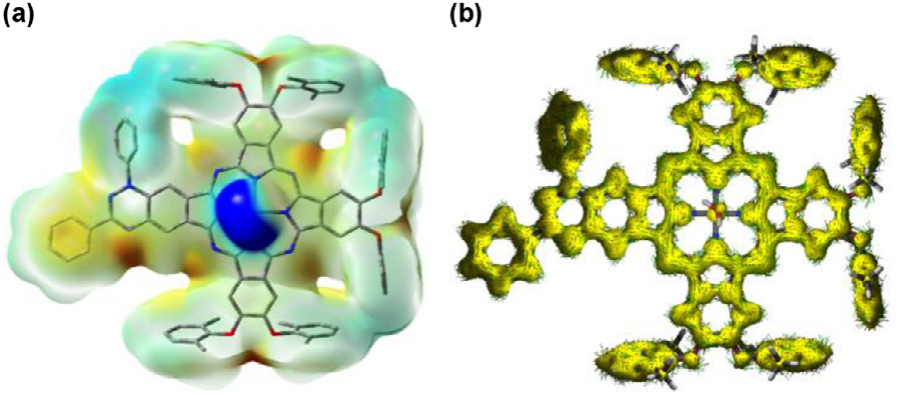
(a) Molecular electrostatic potential (ESP) surface of **ZnPc‐2· B** with the H_2_O ligand, as calculated with UB3LYP/6‐31G(2d,p). The ESP is mapped onto the electron density isosurface, red color indicating an electron‐rich regions and blue color indicating electron‐deficient regions. Green color denotes regions of near‐zero potential, providing a visual reference for electrostatic neutrality across the molecular surface; (b) Anisotropy of the Induced Current Density (ACID) plot of **ZnPc‐2· B** with the H_2_O ligand, as calculated with UB3LYP/6‐31G/NMR (CSGT). The current density is visualized by yellow isosurfaces and green arrows, where the arrows represent the local current density vectors (*J* = 0.05 a.u.).

Electrostatic potential (ESP) mapping (Figures 6a & S25) further supports this electronic partitioning: the most negative ESP regions reside on the TAI unit, while the zinc–aqua moiety defines the most positive region, reflecting its electron‐deficient character. The collective evidence from the ESP, spin density distribution, and frontier orbital separation describes a molecule characterized by a significant intramolecular dipole, polarized from the electron‐deficient zinc‐aqua center and the electron‐rich radical fragment. This ground‐state polarization is a key feature that likely modulates the redox, magnetic, and photophysical properties of the complex.

The anisotropy of the induced current density (ACID) [106, 107] plot of **ZnPc‐2B** (DFT/B3LYP/6‐31G) visualizes the magnetically induced current density vectors on the isosurface, highlighting the flow of electrons under a perpendicular magnetic field (Figure 6b). The phthalocyanine core exhibits a strong, uniform diatropic ring current, reflecting global aromaticity, while the radical spin density, largely confined to the TAI fragment, does not disrupt the macrocyclic current. Peripheral phenyl rings show weaker, localized diatropic currents, confirming their individual aromatic character. Overall, the macrocyclic current highlights extensive *π*‐electron delocalization within the ZnPc core, which stabilizes the radical and preserves the macrocycle's electronic integrity.

### 1.5 UV–Vis Absorption Spectrum Analysis via TD‐DFT

Time‐dependent DFT (TD‐DFT) calculations were performed on **ZnPc‐2·** with an axial H_2_O ligand at the UB3LYP/6‐31G(2d,p) level. Only doublet excited states with 2*S*+1< 2.5‐A and ⟨*S* ^2^⟩ < 1.3 were considered for the main‐text discussion, as higher‐energy states are heavily spin‐contaminated. States with minor quartet contamination (<*S* ^2^> ∼0.85–1.1) are included as partially allowed. Full numerical data for 45 excited states are provided in the Supporting Information.

Computed oscillator strengths range from nearly zero to ∼0.7. The highest‐intensity transitions corresponding to the Q‐band (∼600 nm) and Soret‐band (∼400 nm), are consistent with typical phthalocyanine behavior. The Q‐band transitions involve frontier orbitals localized on the macrocycle: HOMO*α*,*β* → LUMO*α*,*β* (627 nm, *f* = 0.706) and HOMO*α*,*β* → LUMO+1*α*,*β* (598 nm, *f* = 0.391). Radical‐specific SOMO excitations define the low‐energy region: SOMO*α* → LUMO*α* (897 nm, *f* = 0.002), SOMO*α* → LUMO+1*α* (746 nm, *f* = 0.013), and HOMO*β* → SOMO*β* (776 nm, *f* = 0.031), reflecting minimal electronic coupling between the TAI radical and the macrocyclic scaffold.

Soret‐band transitions involve lower‐lying occupied orbitals, representing *π* → π* excitations delocalized over the phthalocyanine macrocycle: HOMO–1/–2/–3*α*,*β* → LUMO/LUMO+1*α*,*β* (432 nm, *f* = 0.211; 400 nm, *f* = 0.107).

**FIGURE 7:**
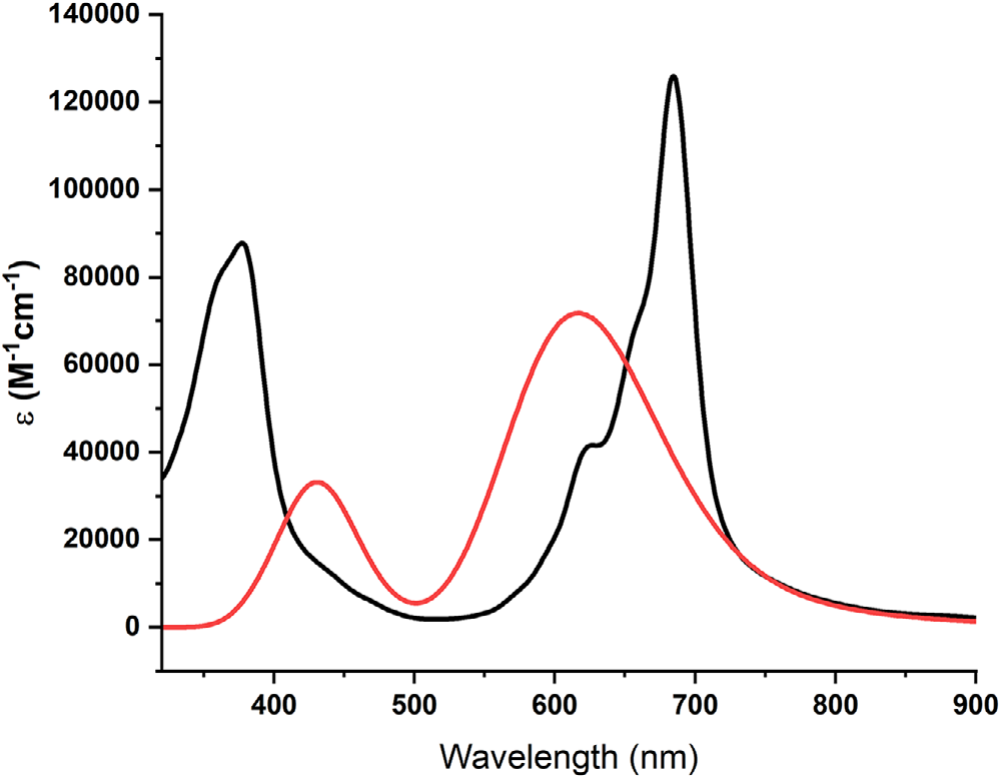
Simulated UV‐vis absorbance spectrum of **ZnPc‐2·** (red) obtained from a UB3LYP/6‐31G(2d,p) TD‐DFT calculation, based on 45 excited states and the experimental UV‐vis absorbance spectrum of **ZnPc‐2·** in DMF (black).

Overall, the TD‐DFT results reproduce the characteristic near‐infrared Q‐band and near‐UV Soret‐band absorptions of phthalocyanines, while highlighting the electronic decoupling of the radical center, in agreement with the FMO and spin density analysis (Figure 7), as well as the experimental spectra. Selected transitions, including radical‐specific SOMO excitations, are summarized in Table 1.

**TABLE 1:** Selected TD‐DFT excited states of **ZnPc‐2B** [UB3LYP/6‐31G(2d,p)]. Radical‐specific SOMO excitations are highlighted; states with minor quartet contamination are labeled as partially allowed.

**Excited** **State**	***E*** **[eV]**	***λ*** **[nm]**	***F***	**<*S* ^2^>**	**Dominant Transition**	**Radical‐specific?**	**Allowed**
3	1.383	897	0.0018	0.852	SOMO*α* → LUMO*α*	Yes	Partially
4	1.598	776	0.0307	1.098	HOMO*β* → SOMO*β*	Yes	Partially
5	1.662	746	0.0126	0.806	SOMO*α* → LUMO+1*α*	Yes	Yes
6	1.979	627	0.7075	0.771	HOMO*α*,*β* → LUMO*α*,*β*	No	Yes
7	2.073	598	0.3910	0.782	HOMO*α*,*β* → LUMO+1*α*,*β*	No	Yes
21	2.844	436	0.0618	0.949	HOMO–1/–2/–3*α*,*β* → LUMO/LUMO+1*α*,*β*	No	Partially
23	2.869	432	0.2107	0.927	HOMO–3*α*,*β* → LUMO*α*,*β*	No	Partially

## 2 Conclusion

We report the first successful synthesis and characterization of a novel class of zinc phthalocyanines, **ZnPc‐1** and **ZnPc‐2**, featuring a *β*‐fused Blatter radical. Structural analysis, anchored by single‐crystal X‐ray diffraction of **ZnPc‐2·**, revealed packing motifs dominated by centrosymmetric *π*‐*π* dimers. Electronic characterization and DFT calculations confirm a stable radical where primary redox events are attributed to the SOMO. Critically, the DFT analysis indicates measurable spin communication between the Blatter unit and the Pc macrocycle, demonstrating that these are cooperative hybrid systems. The resulting molecular architecture, which integrates an open‐shell Blatter radical with a closed‐shell phthalocyanine π‐system, establishes a robust platform for developing materials with tunable spin and optical properties. This system serves as a distinctive model for studying spin–photon interactions in coupled radical–chromophore hybrids.

## Conflicts of Interest

The authors declare no conflict of interest.

## Supporting information



Full experimental procedures, synthesis and characterization of **ZnPc‐1·** and **ZnPc‐2·**, single‐crystal X‐ray diffraction data, electrochemical and spectroscopic analyses, and computational details including DFT and TD‐DFT studies. Deposition Numbers 2482609 and 2482610 contain the supplementary crystallographic data for this paper, available free of charge from the Cambridge Crystallographic Data Centre and Fachinformationszentrum Karlsruhe Access Structures service. **Supporting file 1**: chem70561‐sup‐0001‐SuppMat.docx
