# Supplementary material for: Unsymmetrical β‐Fused Blatter Radical Zinc Phthalocyanines
Source: Chemistry. 2025 Dec 14;32(4):e03294. doi: 10.1002/chem.202503294 (PMC12840835; doi:10.1002/chem.202503294)
Supplement: Supplementary file 1 — Full experimental procedures, synthesis and characterization of ZnPc‐1· and ZnPc‐2·, single‐crystal X‐ray diffraction data, electrochemical and spectroscopic analyses, and computational details including DFT and TD‐DFT studies. Deposition Numbers 2482609 and 2482610 contain the supplementary crystallographic data for this paper, available free of charge from the Cambridge Crystallographic Data Centre and Fachinformationszentrum Karlsruhe Access Structures service. Supporting file 1: chem70561‐sup‐0001‐SuppMat.docx [file CHEM-32-e03294-s001.docx]

Supporting Information
©Wiley-VCH 2021
69451 Weinheim, Germany

Unsymmetrical *β*-Fused Blatter Radical Zinc Phthalocyanines

Adrián Hernández,^[a]^ Georgia A. Zissimou,^[b]^ Javier Ortiz,^[a]^ Andreas Kourtellaris,^[c]^ Christos P. Constantinides,^[d]^ Daniel B. Lawson,^[d]^ Ziqi Hu,^[a]^ Eugenio Coronado,^[e]^ Panayiotis A. Koutentis ^[b],*^ and Ángela Sastre-Santos,^[a],*^

[a] Dr. Adrián Hernández, Dr. Javier Ortiz and Prof. Ángela Sastre-Santos

Área de Química Orgánica, Instituto de Bioingeniería

Universidad Miguel Hernández

Avda. de la Universidad s/n 03203 Elche, Spain

E-mail: [asastre@umh.es](mailto:asastre@umh.es)

[b] Dr. Georgia A. Zissimou, Prof. Panayiotis A. Koutentis
Department of Chemistry
University of Cyprus
P.O. Box 20537, 1678 Nicosia, Cyprus

E-mail: [zissimou.georgia@ucy.ac.cy](mailto:zissimou.georgia@ucy.ac.cy); [koutenti@ucy.ac.cy](mailto:koutenti@ucy.ac.cy)

[c] Dr. Andreas Kourtellaris
Department of Life Sciences, School of Sciences

European University Cyprus

6 Diogenis Str., Engomi, P. O. Box 22006, 1516 Nicosia, Cyprus

E-mail: [A.Kourtellaris@euc.ac.cy](mailto:A.Kourtellaris@euc.ac.cy)

[d] Dr. Christos P. Constantinides and Dr. Daniel B. Lawson

Department of Natural Sciences
University of Michigan – Dearborn
4901 Evergreen Rd, Dearborn, MI 48128, United States

E-mail: [cconst@umich.edu](mailto:cconst@umich.edu); [dblawson@umich.edu](mailto:dblawson@umich.edu)

[e] Prof. Eugenio Coronado

Table of Contents

1. **Experimental Procedures**

**General Materials and Methods**

1. **Synthesis and Characterization of Blatter Radicals**

**Synthesis of ZnPc-1· and ZnPc-2·**

*Figure S1. Molecular structures of phthalonitriles* ***3****-****6****.*

1. **ZnPc-1·**

*Scheme S1. Synthetic Route of* ***ZnPc-1·****.*

*Figure S2. ^1^H NMR of* ***ZnPc-1·*** *in acetone-d*_6_*.*

*Figure S3. ^1^H NMR of* ***ZnPc-1·*** *+ ascorbic acid in acetone-d*_6_*.*

*Figure S4. UV-vis absorption spectra of radical (black) and reduced (red)* ***ZnPc-1·*** *in DMF.*

*Figure S5. FT-IR of* ***ZnPc-1·****.*

*Figure S6. Cyclic voltammogram of* ***ZnPc-1·****.*

*Figure S7. Differential Pulse Voltammetry of* ***ZnPc-1·****.*

*Figure S8. HR-MALDI-TOF spectrum of* ***ZnPc-1·****.*

1. **ZnPc-2·**

*Scheme S2. Synthetic Route of* ***ZnPc-2·****.*

*Figure S9. ^1^H NMR of* ***ZnPc-2·*** *in THF-d*_8_*.*

*Figure S10. ^1^H NMR of* ***ZnPc-2·*** *+ ascorbic acid in THF-d*_8_*.*

*Figure S11. UV-vis absorption spectra of radical (black) and reduced (red)* ***ZnPc-2·*** *in DMF.*

*Figure S12. FT-IR of* ***ZnPc-2·****.*

*Figure S13. Cyclic voltammogram of* ***ZnPc-2·****.*

*Figure S14. Differential Pulse Voltammetry of* ***ZnPc-2****.*

*Figure S15. HR-MALDI-TOF spectrum of* ***ZnPc-2·****.*

Table S1. Electro-Optical properties of the different MPcs.

1. **Single Crystal X–ray Diffractometry Studies of ZnPc-2· A and B**

**X-ray methodology**

**Structure of ZnPc-2· A (CCDC 2482610)**

Table S2. Crystal data and structure refinement for **ZnPc-2· A**

Figure S16. ORTEP view of the phthalocyanine radical **ZnPc-2· A**

Table S3. Selected crystallographic bond lengths and bond angles of phthalocyanine radical **ZnPc-2· A**

**Structure of ZnPc-2· B (CCDC 2482609)**

Table S4. Crystal data and structure refinement for **ZnPc-2· B**.

Figure S17. ORTEP view of the phthalocyanine radical **ZnPc-2· B**

Table S5. Selected crystallographic bond lengths and bond angles of phthalocyanine radical **ZnPc-2· B**

Table S6. Deviations of benzo ring centroids (yellow) and maximum atomic displacements.

Table S7. Deviations of individual atoms from the 13-atom mean plane.

Figure S18. *PorphyStruct* results for **ZnPc-2·** radicals **A** and **B**.

Figure S19. Relative orientations of adjacent **ZnPc-2·** molecules **A** and **B** within the herringbone motif and unit cell.

Figure S20. Crystal packing of **ZnPc-2·** molecules A and B in a 2×2×2 unit cell expansion.

Table S8. *Cis*-oriented torsion angles (*Φ*, °) of the triazinyl–phenyl substituents in **ZnPc-2· A** and **B**.

**Secondary and solvent interactions**

Table S9. Solvent and secondary interactions within phthalocyanine radicals **ZnPc-2· A** and **B**.

1. **Computational Studies**

**Computational methodology**

**Further discussion**

Table S10. Computational results for **ZnPc-2· A** and **B** radicals.

Table S11. Selected TD-DFT excited states of **ZnPc-2· A**.

Table S12. Selected TD-DFT excited states of **ZnPc-2· B**.

Figure S21. Simulated UV-vis spectrum of **ZnPc-2· A** and **B**.

Figure S22. Dipole vector of **ZnPc-2· B**.

Figure S23. Spin density map of **ZnPc-2· B**.

Figure S24. Molecular electrostatic potential (ESP) surface of **ZnPc-2· B**.

Figure S25. Frontier molecular orbital (FMOs) surfaces for **ZnPc-2· B**.

**Output data from TD-DFT calculations at UB3LYP/6-31G(2d,p)**

1. **References**

# Experimental Procedures

## General Materials and Methods

All reagents and solvents were purchased from Sigma Aldrich (Merck) and TCI, and were used as received unless otherwise stated. All volatiles were removed under reduced pressure. Microwave-assisted reactions were carried out in a Discover SP reactor (CEM Corporation). Product purification by flash chromatography was performed on a CombiFlash® NextGen 300 system. NMR spectra were recorded on a BRUKER AVANCE NEO 400 spectrometer. UV-vis spectra were measured with a Perkin-Elmer Lambda 365 spectrophotometer, while fluorescence spectra were obtained using a HORIBA Scientific SAS spectrophotometer. High-resolution mass spectra were collected on a Bruker Microflex LRF20 MALDI-TOF instrument, employing dithranol as matrix. Infrared spectra were acquired with a Nicolet Impact 400D spectrophotometer.

1,3-Diphenyl-1,4-dihydrobenzo[*e*][1,2,4]triazine-6,7-dicarbonitrile (**3**),**^1^** 1,3-diphenyl-1,4-dihydrobenzo-[*e*][1,2,4]triazine-6,7-dicarbonitrile (**4**),**^1^** 4,5-bis[4-(2,4,4-trimethylpentan-2-yl)phenoxy]phthalonitrile (**5**),**^2^** and 4,5-bis(2,6-dimethylphenoxy)phthalonitrile (**6**)**^3^** were prepared according to the literature.

Cyclic voltammetry measurements were conducted in a conventional three-electrode setup with a μ-AUTOLAB type III potentiostat/galvanostat at 298 K in DMF, using deaerated solutions containing 0.10 M tetrabutylammonium hexafluorophosphate (TBAPF_6_) as supporting electrolyte. Platinum was employed as the working electrode, Ag/AgNO_3_ (0.01 M in benzonitrile) as the reference electrode, and a platinum wire as counter electrode. The ferrocene/ferrocenium redox couple was used as internal standard for all measurements.

# Synthesis and Characterization of Blatter Radicals

## Synthesis of ZnPc-1· and ZnPc-2·

**Figure S1.** Molecular structures of phthalonitriles **3-6**.

## ZnPc-1·

**Scheme S1**. Synthetic Route of **ZnPc-1·**.

A stirred suspension of 1,3-diphenyl-1,4-dihydrobenzo[*e*][1,2,4]triazine-6,7-dicarbonitrile (**3**) (20.0 mg, 0.06 mmol), 4,5-bis[4-(2,4,4-trimethylpentan-2-yl)phenoxy]phthalonitrile (**5**) (100.0 mg, 0.19 mmol) and Zn(OAc)_2_ (22.0 mg, 0.12 mmol) in dimethylethanolamine (DMAE, 500 µL) in a sealed vial, under inert atmosphere, was inserted in a CEM Discovery microwave reactor and irradiated (100 W) to *ca*. 145 °C for 90 min at *ca.* 15 PSI (0.1 MPa). Upon completion, the reaction vessel was air cooled to *ca*. 20 °C, the solvent was concentrated under vacuum and the mixture was purified by column chromatography (SiO_2_, CHCl_3_/EtOAc, 96:4), followed up by MeOH washings (3 × 2 mL, centrifuge 10 min at 400 rpm) to give the *titled compound* **ZnPc-1·** (15.0 mg, 13%) as a green solid. *m/z* (HR-MALDI-TOF/dithranol) for C_129_H_144_N_11_O_6_Zn·: calcd 2007.059 [M^+^]; found, 2006.966; *δ*_H_ (400 MHz, Acetone-*d*_6_ & ascorbic acid) 0.83–0.91 (m, 54H), 1.32–1.44 (m, 38H), 1.84–1.88 (d, *J* = 13.0 Hz, 11H), 6.71 (s, 1H), 7.20–7.70 (m, 33H), 7.98–8.07 (m, 2H), 8.17(s, 2H), 8.36 (s, 2H), 8.51 (s, 1H), 8.65 (s, 1H), 8.83 (s, 1H), 8.94–9.01 (d, 2H), 9.72 (s, 2H); *λ*_max_(DMF)/nm (log *ε*) 376 (5.05), 624 (4.72), 685, (5.27); *λ*_max_ (DMF+ascorbic acid)/nm (log *ε*) 364 (5.07), 618 (4.72), 684 (5.33); *ν*_max_/cm^-1^ 3038, 2953, 2903, 1603, 1506, 1448, 1403, 1365, 1271, 1217, 1179, 1089, 1030, 891, 829, 747, 693, 581, 511, 453.

**Figure S2.** ^1^H NMR of **ZnPc-1·** in acetone-d_6_.

**Figure S3.** ^1^H NMR of **ZnPc-1·** + ascorbic acid in acetone-d_6_.

**Figure S4.** UV-vis absorption spectra of radical (black) and reduced (red) **ZnPc-1·** in DMF.

**Figure S5.** FT-IR of **ZnPc-1·**.


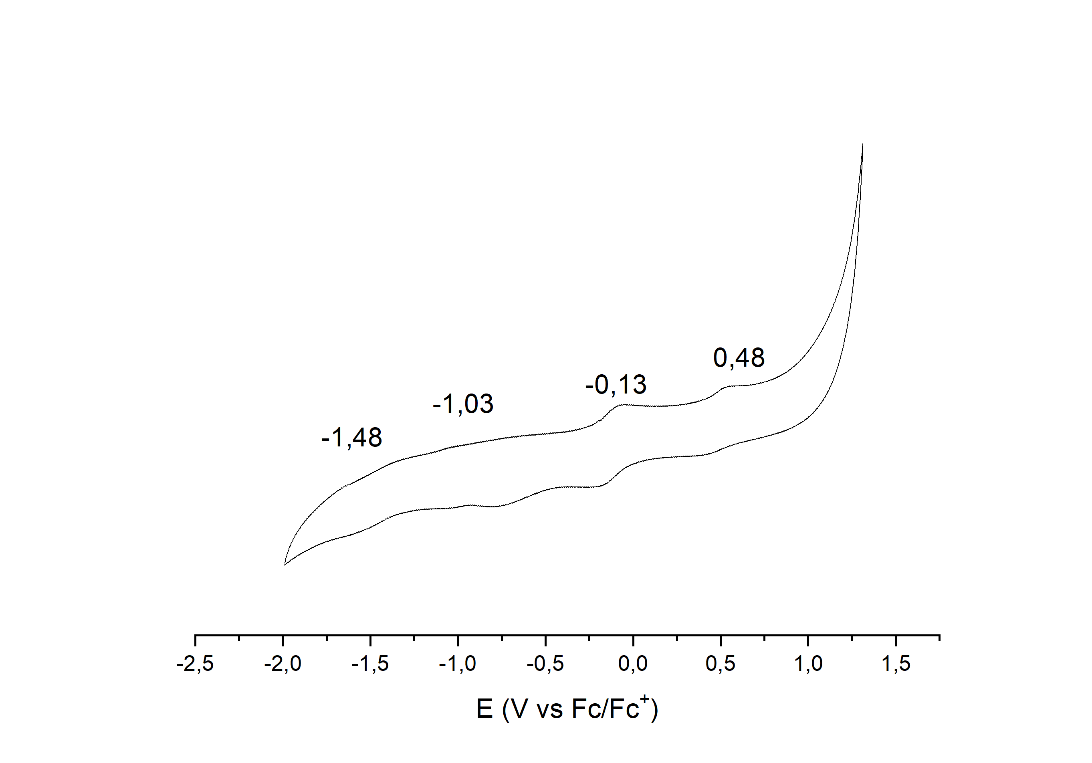


**Figure S6.** Cyclic voltammogram of **ZnPc-1·**.


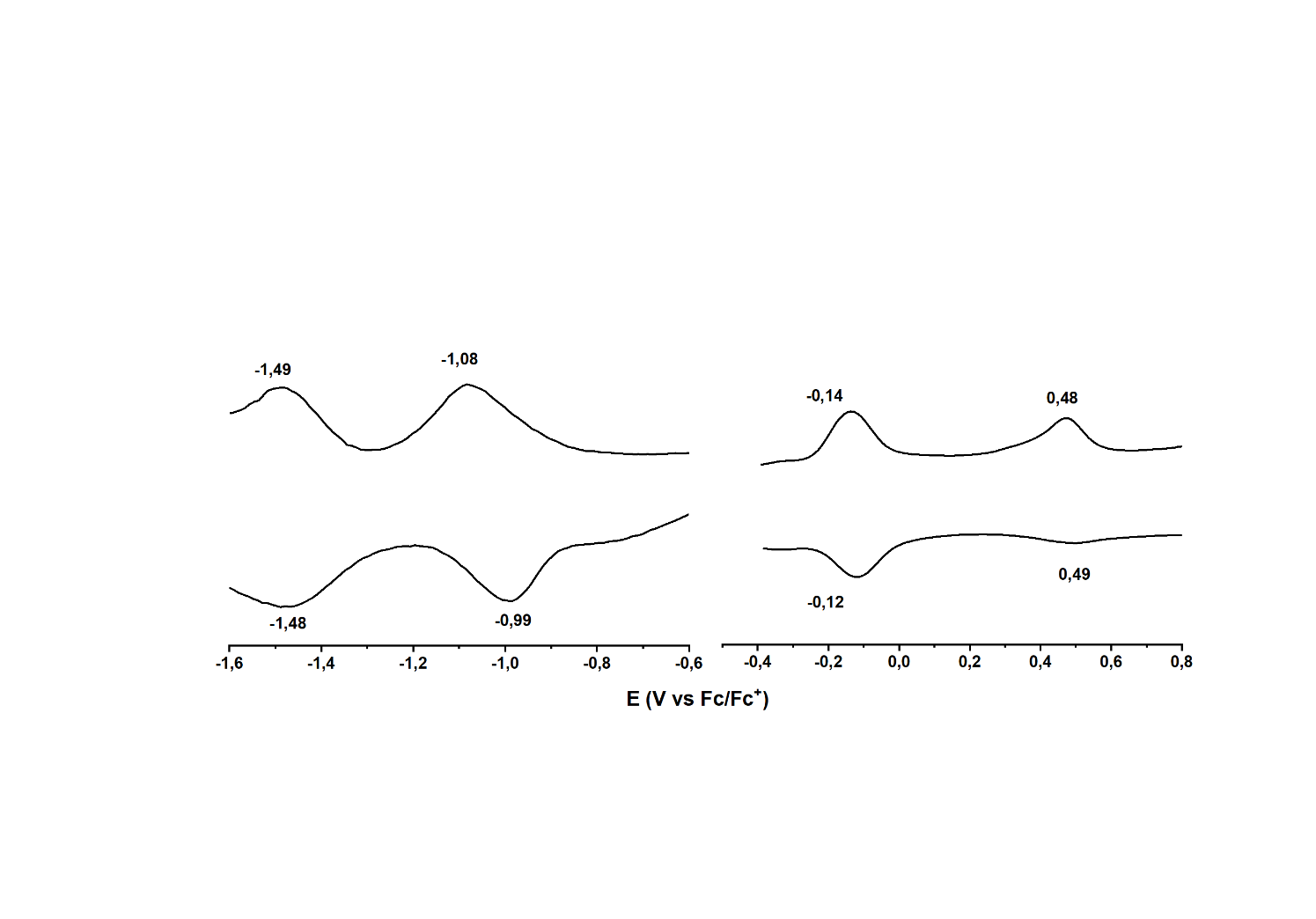


**Figure S7.** Differential Pulse Voltammetry of **ZnPc-1·**.


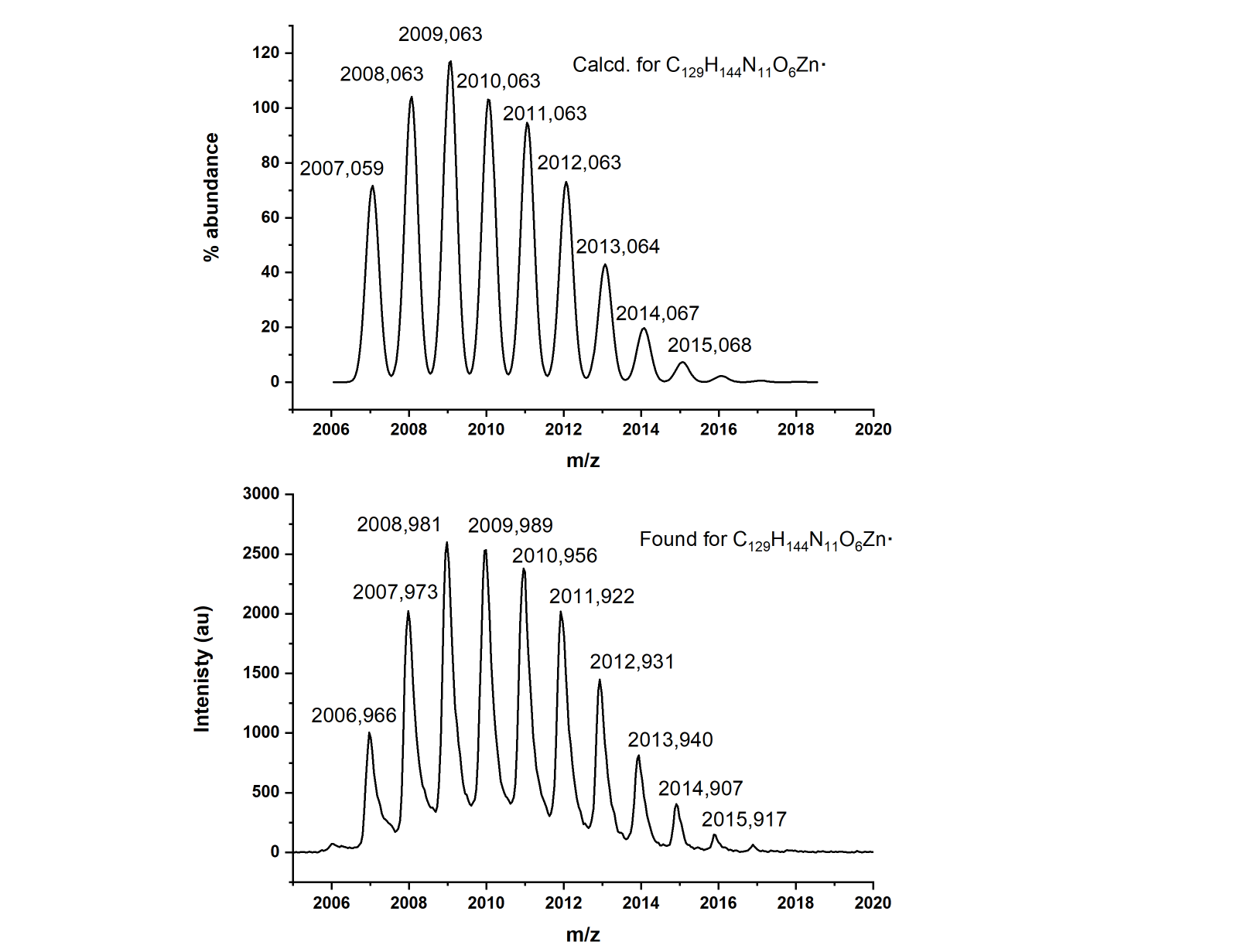


**Figure S8.** HR-MALDI-TOF spectrum of **ZnPc-1·**.

## ZnPc-2·

**Scheme S2.** Synthetic Route of **ZnPc-2·**.

A stirred suspension of 1,3-diphenyl-1,4-dihydrobenzo[*e*][1,2,4]triazin-4-yl-6,7-dicarbonitrile (**4**) (30.0 mg, 0.09 mmol), 4,5-bis(2,6-dimethylphenoxy)phthalonitrile (**6**) (105.0 mg, 0.28 mmol) and Zn(OAc)_2_ (33.0 mg, 0.18 mmol) in dimethylethanolamine (DMAE, 500 µL) in a sealed vial, under inert atmosphere, was inserted in a CEM Discovery microwave reactor and irradiated (100 W) to *ca*. 145 °C for 90 min at *ca.* 15 PSI (0.1 MPa). Upon completion, the reaction vessel was air cooled to *ca*. 20 °C, the solvent was concentrated under vacuum and the mixture was purified by column chromatography (SiO_2_, CHCl_3_/EtOAc, 96:4), followed up by MeOH washings (3 × 2 mL, centrifuge 10 min at 400 rpm) to give the *titled compound* **ZnPc-2·** (22.0 mg, 16%) as a green solid. *m/z* (HR-MALDI-TOF/dithranol) for C_93_H_72_N_11_O_6_Zn· calcd 1502.495 [M^+^]; found, 1502.403; *δ*_H_ (400 MHz, THF-*d*_8_ & ascorbic acid) 2.41–2.43 (m, 37H), 7.34–7.60 (m, 24H), 7.81–7.83 (d, *J* = 7.4 Hz, 2H), 7.97–8.00 (m, 2H), 8.10–8.12 (m, 3H), 8.17–8.18 (d, *J* = 4.6 Hz, 2H), 8.20 (s, 1H), 8.32 (s, 1H), 8.60 (s, 1H), 9.85 (s, 4H); *λ*_max_(DMF)/nm (log *ε*) 378 (4.94), 628 (4.61), 653 (4.80), 685 (5.10); *λ*_max_(DMF+ascorbic acid)/nm (log *ε*) 361 (4.98), 615 (4.59), 682 (5.22); *ν*_max_/cm^-1^ 2922, 1587, 1450, 1400, 1342, 1273, 1186, 1092, 1028, 891, 766, 692, 509.

**Figure S9.** ^1^H NMR of **ZnPc-2·** in THF-d_8_.

**Figure S10.** ^1^H NMR of **ZnPc-2·** + ascorbic acid in THF-d_8_.

**Figure S11.** UV-vis absorption spectra of radical (black) and reduced (red) **ZnPc-2·** in DMF.

**Figure S12.** FT-IR of **ZnPc-2·**.


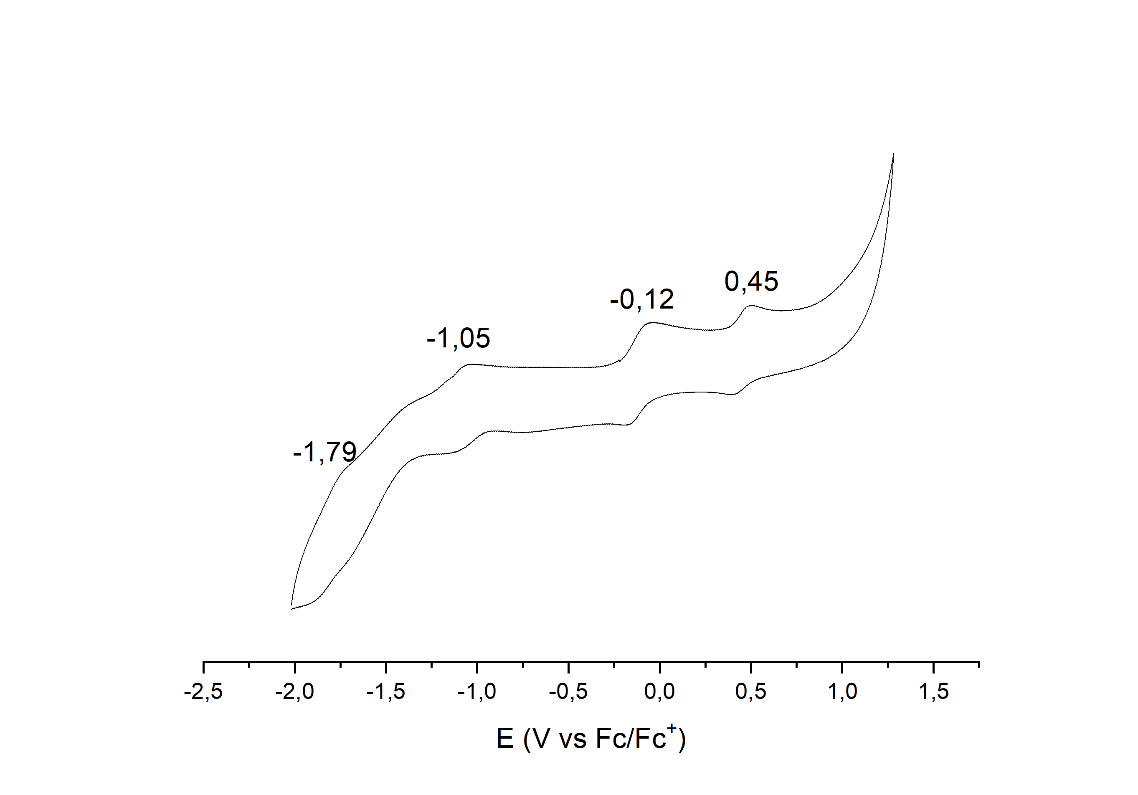


**Figure S13.** Cyclic voltammogram of **ZnPc-2·**.


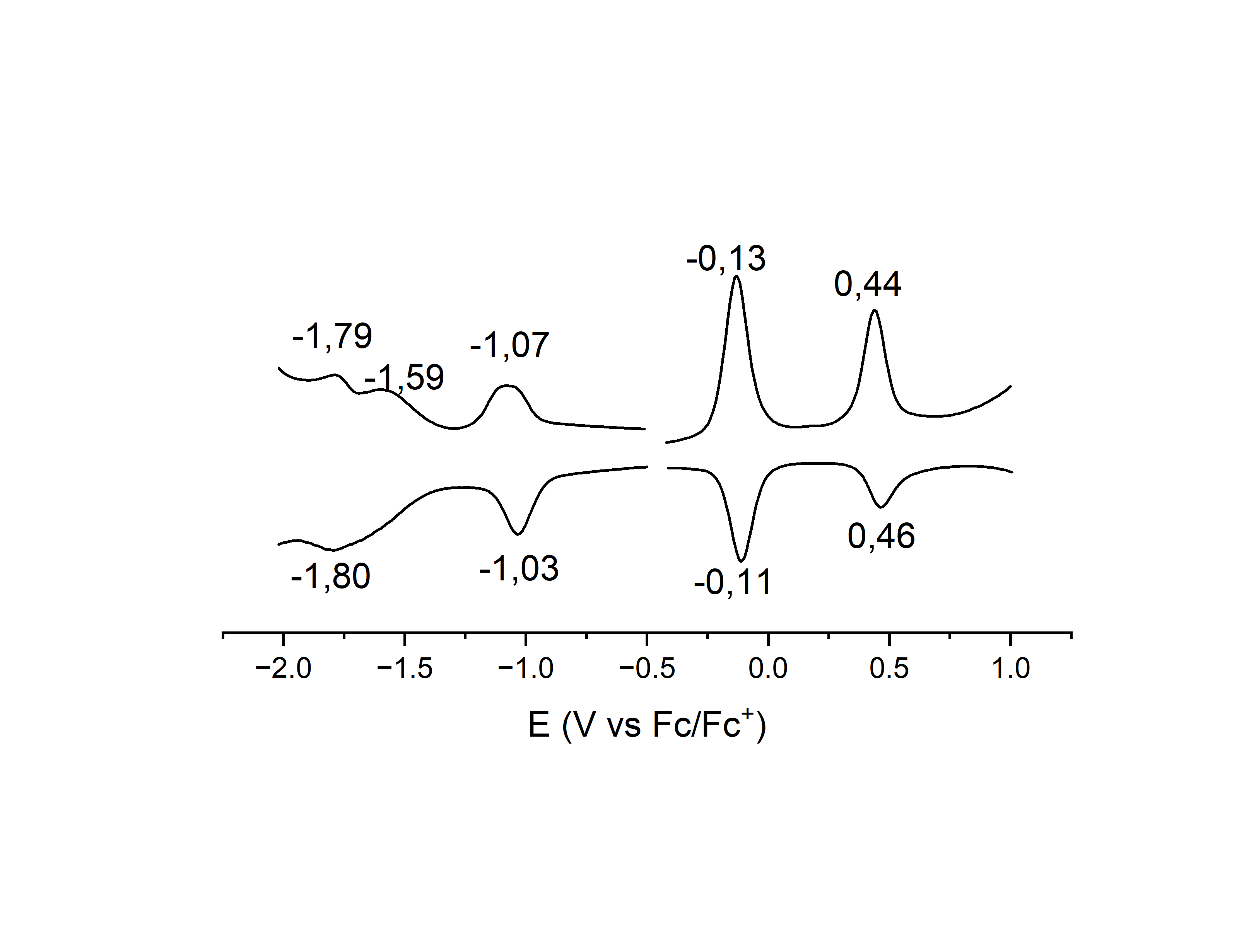


**Figure S14.** Differential Pulse Voltammetry of **ZnPc-2·**.

**Figure S15.** HR-MALDI-TOF spectrum of **ZnPc-2·**.

Singly occupied molecular orbital energy levels (*E*_SOMO_) were calculated using the equation *E*_SOMO_ = −4.8 − *E*_Ox1_, where *E*_Ox1_ represents the first oxidation potential. For **ZnPc-1·** the SOMO energy level (*E*_SOMO_) was found at −4.67 eV and for **ZnPc-2·** at −4.68 eV. The LUMO energy level (*E*_LUMO_) was determined by adding the band gap to the *E*_SOMO_ value, resulting in −3.77 eV and −3.75 eV for **ZnPc-1·** and **ZnPc-2·**, respectively (Table S1).

**Table S1.** Electro-Optical properties of the different MPcs.

| MPc | Q band  λ_max_/nm | $\boldsymbol{E}_{\boldsymbol{g}}^{\mathbf{EQ}}$  (V) | *E*_Ox1_  (V) | *E*_Ox2_  (V) | *E*_Red1_  (V) | *E*_Red2_  (V) | *E*_SOMO_  (eV) | *E*_LUMO_  (eV) |
| --- | --- | --- | --- | --- | --- | --- | --- | --- |
| ZnPc-1· | 685 | 0.90 | -0.13 | 0.48 | -1.03 | -1.48 | -4.67 | -3.77 |
| ZnPc-2· | 685 | 0.93 | -0.12 | 0.45 | -1.05 | -1.79 | -4.68 | -3.75 |

# Single Crystal X–ray Diffractometry Studies of ZnPc-2· A and B

## X-ray methodology

Data were collected on an XtaLAB Synergy, Single source at home/near, HyPix diffractometer equipped with a CCD area detector utilizing Cu-K*α* radiation (*λ* = 1.5418 Å). Suitable crystals were attached to glass fibers using paratone-N oil and transferred to a goniostat, where they were cooled for data collection. Unit cell dimensions were determined and refined by using 108915 (2.59 ≤ *θ* ≤ 75.72°) reflections for **ZnPc-2· A** and 118959 (2.62 ≤ *θ* ≤ 75.80°) reflections for **ZnPc-2· B**. Empirical absorption corrections (multi-scan based on symmetry-related measurements) were applied using CrysAlis RED software.**^4^** The structures were solved using Olex2,**^5^** with the olex2.solve**^6^** structure solution program using Charge Flipping and refined with the SHELXL**^7^** refinement package using Least Squares minimization. Mercury**^8^** was used for molecular graphics. The non-H atoms were treated anisotropically. Hydrogen atoms were placed in calculated positions and refined using a riding model, except for those attached to [O1], which were located from difference Fourier maps and refined with restrained distances. *PorphyStruct***^9^** was used to generate non-planar conformation graphs.

## Structure of ZnPc-2· A (CCDC 2482610)

**Table S2.** Crystal data and structure refinement for **ZnPc-2· A**, C_98_ H_84_ Br_5_ N_11_ O_7_ Zn at 100(2) K.

| Empirical formula | C_98_ H_84_ Br_5_ N_11_ O_7_ Zn |
| --- | --- |
| Formula weight | 1992.68 |
| Temperature | 100(2) K |
| Wavelength | 1.54184 Å |
| Crystal system | Monoclinic |
| Space group | *P* 2_1_/*c* |
| Unit cell dimensions | *a* = 11.9954(2) Å, *α* = 90°  *b* = 34.1212(6) Å, *β* = 99.507(2)°  *c* = 24.5199(5) Å, *γ* = 90° |
| Volume | 9898.1(3) Å^3^ |
| *Z* | 4 |
| Density (calculated) | 1.337 g/cm^3^ |
| Absorption coefficient | 3.160 mm^-1^ |
| *F*(000) | 4040 |
| Crystal size | 0.174 × 0.025 × 0.015 mm^3^ |
| *θ* range for data collection | 2.590 to 75.721° |
| Index ranges | -8 < = h < = 14, -42 < = k < = 40, -30 < = l < = 30 |
| Reflections collected | 108915 |
| Independent reflections | 19872 [*R*_int_ = 0.0991] |
| Completeness to *θ* = 67.684° | 99.4% |
| Refinement method | Full-matrix least-squares on *F*^2^ |
| Data / restraints / parameters | 19872 / 1837 / 1118 |
| Goodness-of-fit | 1.113 |
| Final R indices [*I* > 2*σ*(*I*)] | *R*_obs_ = 0.1116, *wR*_obs_ = 0.3208 |
| R indices [all data] | *R*_all_ = 0.1450, *wR*_all_ = 0.3515 |
| Largest diff. peak and hole | 1.417 and -1.970 e·Å^-3^ |

*R* = *Σ*||*F*_o_|-|*F*_c_||/*Σ*|*F*_o_|, *wR* = {*Σ*[*w*(|*F*_o_|^2^-|*F*_c_|^2^)^2^]/*Σ*[*w*(|*F*_o_|^4^)]}^1/2^ and *w* = 1/[*σ*^2^(*F*_o_^2^)+(0.1981*P*)^2^+26.8228*P*] where *P* = (*F*_o_^2^+2*F*_c_^2^)/3


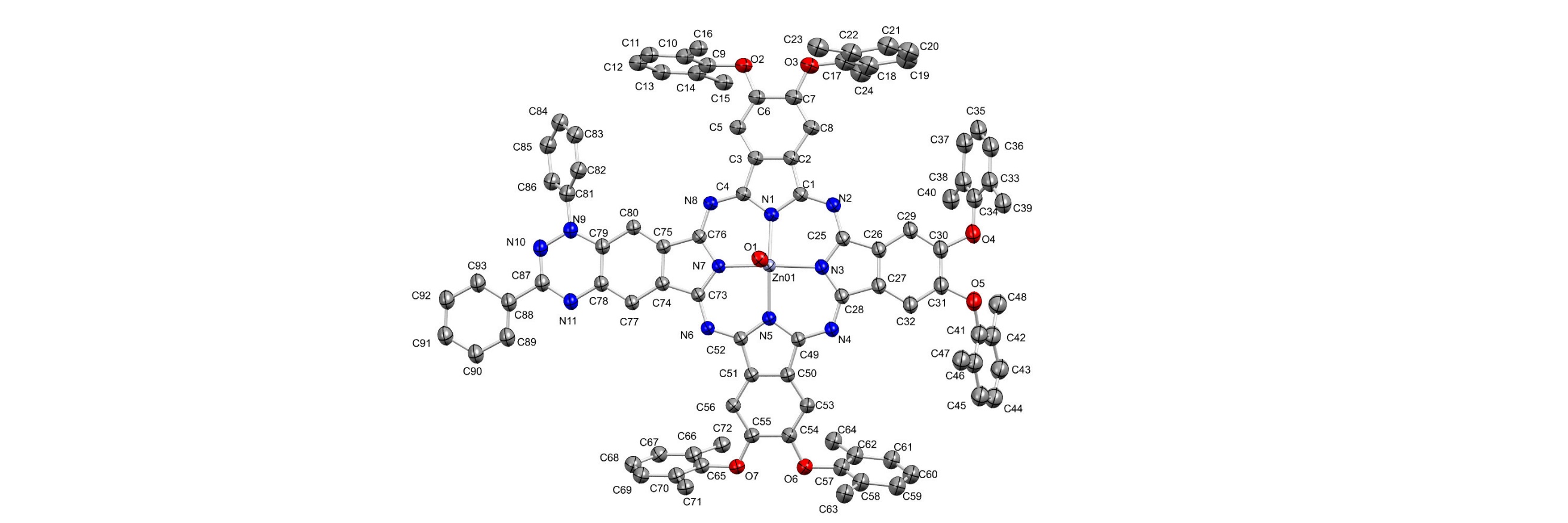


**Figure S16.** ORTEP view of the phthalocyanine radical **ZnPc-2· A** (CCDC 2482610) with thermal ellipsoid model at 50% probability level. Solvent molecules and hydrogen atoms omitted for clarity. Crystallographic numbering shown.

**Table S3.** Selected crystallographic bond lengths and bond angles of phthalocyanine radical **ZnPc-2· A** (CCDC 2482610).

| Bond Lengths (Å) | | | Bond Angles (°) | | | |
| --- | --- | --- | --- | --- | --- | --- |
| N9 | N10 | 1.366(9) | C81 | N9 | N10 | 113.5(6) |
| N10 | C87 | 1.33(1) | C81 | N9 | C79 | 123.4(6) |
| C87 | N11 | 1.34(1) | C79 | N9 | N10 | 122.9(6) |
| N11 | C78 | 1.365(9) | N9 | N10 | C87 | 116.6(6) |
| C78 | C79 | 1.43(1) | N10 | C87 | N11 | 126.2(7) |
| C79 | N9 | 1.40(1) | C87 | N11 | C78 | 117.5(6) |
| C79 | C80 | 1.40(1) | N11 | C78 | C79 | 121.2(6) |
| C78 | C77 | 1.40(1) | C78 | C79 | N9 | 115.6(6) |
| C77 | C74 | 1.384(9) | C80 | C79 | C78 | 122.6(7) |
| C74 | C75 | 1.42(1) | C79 | C78 | C77 | 119.9(6) |
| C75 | C80 | 1.40(1) | C78 | C77 | C74 | 117.6(6) |
| C75 | C76 | 1.443(9) | C77 | C74 | C75 | 122.3(6) |
| C74 | C73 | 1.461(9) | C74 | C75 | C80 | 121.1(6) |
| C73 | N7 | 1.354(9) | C75 | C80 | C79 | 116.4(6) |
| N7 | C76 | 1.371(9) | C76 | C75 | C74 | 106.3(6) |
| C76 | N8 | 1.348(9) | C75 | C74 | C73 | 106.1(6) |
| N8 | C4 | 1.308(9) | C74 | C73 | N7 | 108.7(6) |
| C4 | N1 | 1.37(1) | C73 | N7 | C76 | 109.8(5) |
| N1 | C1 | 1.357(9) | N7 | C76 | C75 | 109.0(6) |
| C1 | N2 | 1.329(9) | C73 | N6 | C52 | 123.7(6) |
| N2 | C25 | 1.34(1) | C76 | N8 | C4 | 123.8(6) |
| C25 | N3 | 1.359(9) | C1 | N2 | C25 | 123.9(7) |
| N3 | C28 | 1.35(1) | C28 | N4 | C49 | 123.7(7) |
| C28 | N4 | 1.331(9) | C49 | N5 | C52 | 108.4(6) |
| N4 | C49 | 1.339(9) | N5 | C52 | C51 | 109.4(6) |
| C49 | N5 | 1.372(8) | C52 | C51 | C50 | 105.8(6) |
| N5 | C52 | 1.355(9) | C51 | C50 | C49 | 107.5(6) |
| C52 | N6 | 1.334(8) | C50 | C49 | N5 | 108.8(6) |
| N6 | C73 | 1.331(9) | C28 | N3 | C25 | 108.9(6) |
| C4 | C3 | 1.46(1) | C25 | C26 | C27 | 106.7(6) |
| C3 | C2 | 1.38(1) | C26 | C27 | C28 | 106.0(6) |
| C2 | C1 | 1.46(1) | C27 | C28 | N3 | 109.0(6) |
| C25 | C26 | 1.444(9) | C26 | C25 | N3 | 109.5(6) |
| C26 | C27 | 1.38(1) | C1 | N1 | C4 | 109.2(6) |
| C27 | C28 | 1.47(1) | N1 | C4 | C3 | 108.0(6) |
| C49 | C50 | 1.43(1) | C4 | C3 | C2 | 107.7(6) |
| C50 | C51 | 1.384(9) | C3 | C2 | C1 | 105.7(6) |
| C51 | C52 | 1.46(1) | C2 | C1 | N1 | 109.3(6) |
| N3 | Zn01 | 2.024(6) | N1 | C4 | N8 | 128.4(7) |
| Zn01 | N1 | 2.023(7) | N8 | C76 | N7 | 127.9(6) |
| Zn01 | N7 | 2.046(5) | N7 | C73 | N6 | 127.7(6) |
| Zn01 | N5 | 2.042(6) | N6 | C52 | N5 | 127.9(6) |
| Zn01 | O1*^a^* | 2.089(5) | N5 | C49 | N4 | 127.3(7) |
|  |  |  | N4 | C28 | N3 | 128.2(7) |
|  |  |  | N3 | C25 | N2 | 127.6(7) |
|  |  |  | N3 | Zn01 | N1 | 87.9(3) |
|  |  |  | N1 | Zn01 | N7 | 87.9(2) |
|  |  |  | N7 | Zn01 | N5 | 86.8(2) |
|  |  |  | N5 | Zn01 | N3 | 87.5(2) |
|  |  |  | O1*^a^* | Zn01 | N7 | 99.9(2) |
|  |  |  | O1*^a^* | Zn01 | N5 | 104.5(2) |
|  |  |  | O1*^a^* | Zn01 | N3 | 104.3(2) |
|  |  |  | O1*^a^* | Zn01 | N1 | 99.1(2) |
|  |  |  | N1 | Zn01 | N5 | 156.3(2) |
|  |  |  | N3 | Zn01 | N7 | 155.8(2) |

*^a^* O1 = aqua oxygen (H1A,H1B)

## Structure of ZnPc-2· B (CCDC 2482609)

**Table S4.** Crystal data and structure refinement for **ZnPc-2· B,** C_107_ H_102_ Br_2_ N_11_ O_7_ Zn at 100(2) K.

| Empirical formula | C_107_ H_102_ Br_2_ N_11_ O_7_ Zn |
| --- | --- |
| Formula weight | 1879.18 |
| Temperature | 100(2) K |
| Wavelength | 1.54184 Å |
| Crystal system | Monoclinic |
| Space group | *P* 2_1_/*n* |
| Unit cell dimensions | *a* = 24.1175(4) Å, *α* = 90°  *b* = 15.6717(3) Å, *β* = 98.465(2)°  *c* = 27.8707(4) Å, *γ* = 90° |
| Volume | 10419.3(3) Å^3^ |
| *Z* | 4 |
| Density (calculated) | 1.198 g/cm^3^ |
| Absorption coefficient | 1.674 mm^-1^ |
| *F*(000) | 3908 |
| Crystal size | 0.313 × 0.119 × 0.018 mm^3^ |
| *θ* range for data collection | 2.622 to 75.798° |
| Index ranges | -30 < = h < = 29, -19 < = k < = 16, -35 < = l < = 34 |
| Reflections collected | 118959 |
| Independent reflections | 21307 [*R*_int_ = 0.0288] |
| Completeness to *θ* = 67.684° | 99.7% |
| Refinement method | Full-matrix least-squares on *F*^2^ |
| Data / restraints / parameters | 21307 / 953 / 1191 |
| Goodness-of-fit | 1.063 |
| Final R indices [*I* > 2*σ*(*I*)] | *R*_obs_ = 0.1127, *wR*_obs_ = 0.3204 |
| R indices [all data] | *R*_all_ = 0.1251, *wR*_all_ = 0.3354 |
| Largest diff. peak and hole | 1.707 and -1.687 e·Å^-3^ |

*R* = *Σ*||*F*_o_|-|*F*_c_||/*Σ*|*F*_o_|, *wR* = {*Σ*[*w*(|*F*_o_|^2^-|*F*_c_|^2^)^2^]/*Σ*[*w*(|*F*_o_|^4^)]}^1/2^ and *w* = 1/[*σ*^2^(*F*_o_^2^)+(0.2000*P*)^2^+30.0000*P*] where *P* = (*F*_o_^2^+2*F*_c_^2^)/3

**
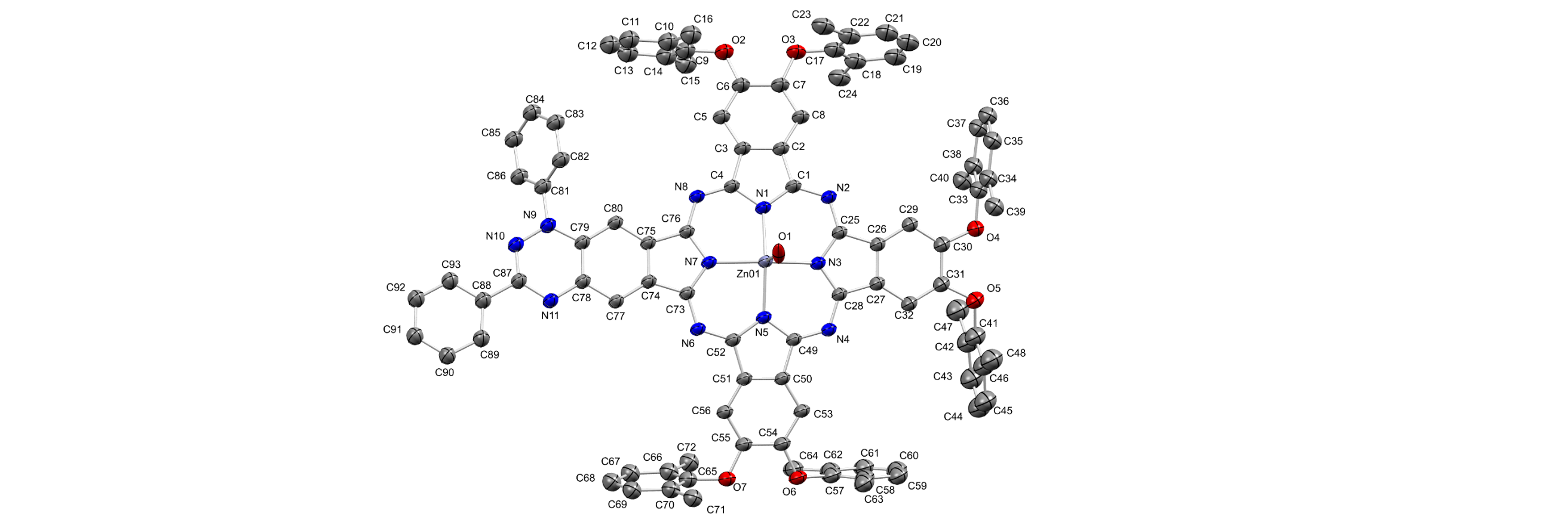
**

**Figure S17.** ORTEP view of the phthalocyanine radical **ZnPc-2· B** (CCDC 2482609) with thermal ellipsoid model at 50% probability. Solvent molecules and hydrogen atoms omitted for clarity. Crystallographic numbering shown.

**Table S5.** Selected crystallographic bond lengths and bond angles of phthalocyanine radical **ZnPc-2· B** (CCDC 2482609).

| **Bond Lengths (Å)** | | | **Bond Angles (°)** | | | |
| --- | --- | --- | --- | --- | --- | --- |
| N9 | N10 | 1.358(5) | C81 | N9 | N10 | 114.1(4) |
| N10 | C87 | 1.327(6) | C81 | N9 | C79 | 122.9(4) |
| C87 | N11 | 1.342(6) | C79 | N9 | N10 | 122.7(4) |
| N11 | C78 | 1.371(5) | N9 | N10 | C87 | 117.0(4) |
| C78 | C79 | 1.421(6) | N10 | C87 | N11 | 126.1(4) |
| C79 | N9 | 1.380(6) | C87 | N11 | C78 | 116.8(4) |
| C79 | C80 | 1.408(6) | N11 | C78 | C79 | 120.9(4) |
| C78 | C77 | 1.399(6) | C78 | C79 | N9 | 116.3(4) |
| C77 | C74 | 1.391(6) | C80 | C79 | C78 | 121.2(4) |
| C74 | C75 | 1.402(6) | C79 | C78 | C77 | 120.3(4) |
| C75 | C80 | 1.384(6) | C78 | C77 | C74 | 118.1(4) |
| C75 | C76 | 1.457(6) | C77 | C74 | C75 | 121.0(4) |
| C74 | C73 | 1.445(6) | C74 | C75 | C80 | 122.2(4) |
| C73 | N7 | 1.367(5) | C75 | C80 | C79 | 117.1(4) |
| N7 | C76 | 1.367(6) | C76 | C75 | C74 | 106.0(4) |
| C76 | N8 | 1.338(5) | C75 | C74 | C73 | 107.0(4) |
| N8 | C4 | 1.320(6) | C74 | C73 | N7 | 108.8(4) |
| C4 | N1 | 1.369(6) | C73 | N7 | C76 | 109.1(4) |
| N1 | C1 | 1.364(5) | N7 | C76 | C75 | 108.9(4) |
| C1 | N2 | 1.335(6) | C73 | N6 | C52 | 123.4(4) |
| N2 | C25 | 1.334(6) | C76 | N8 | C4 | 123.4(4) |
| C25 | N3 | 1.365(5) | C1 | N2 | C25 | 123.3(4) |
| N3 | C28 | 1.366(6) | C28 | N4 | C49 | 122.8(4) |
| C28 | N4 | 1.333(6) | C49 | N5 | C52 | 108.5(3) |
| N4 | C49 | 1.331(5) | N5 | C52 | C51 | 108.9(4) |
| C49 | N5 | 1.364(6) | C52 | C51 | C50 | 106.3(4) |
| N5 | C52 | 1.379(5) | C51 | C50 | C49 | 106.9(4) |
| C52 | N6 | 1.331(6) | C50 | C49 | N5 | 109.3(4) |
| N6 | C73 | 1.343(6) | C28 | N3 | C25 | 108.6(4) |
| C4 | C3 | 1.464(6) | C25 | C26 | C27 | 106.1(4) |
| C3 | C2 | 1.388(7) | C26 | C27 | C28 | 106.4(4) |
| C2 | C1 | 1.453(6) | C27 | C28 | N3 | 109.6(4) |
| C25 | C26 | 1.458(6) | C26 | C25 | N3 | 109.2(4) |
| C26 | C27 | 1.405(6) | C1 | N1 | C4 | 109.1(4) |
| C27 | C28 | 1.446(6) | N1 | C4 | C3 | 108.6(4) |
| C49 | C50 | 1.455(6) | C4 | C3 | C2 | 106.4(4) |
| C50 | C51 | 1.394(6) | C3 | C2 | C1 | 106.9(4) |
| C51 | C52 | 1.462(6) | C2 | C1 | N1 | 109.0(4) |
| N3 | Zn01 | 2.018(4) | N1 | C4 | N8 | 128.1(4) |
| Zn01 | N1 | 2.021(4) | N8 | C76 | N7 | 127.9(4) |
| Zn01 | N7 | 2.038(4) | N7 | C73 | N6 | 127.7(4) |
| Zn01 | N5 | 2.026(3) | N6 | C52 | N5 | 127.2(4) |
| Zn01 | O1*^a^* | 2.156(5) | N5 | C49 | N4 | 129.0(4) |
|  |  |  | N4 | C28 | N3 | 127.4(4) |
|  |  |  | N3 | C25 | N2 | 127.8(4) |
|  |  |  | N3 | Zn01 | N1 | 88.0(2) |
|  |  |  | N1 | Zn01 | N7 | 87.4(2) |
|  |  |  | N7 | Zn01 | N5 | 86.8(1) |
|  |  |  | N5 | Zn01 | N3 | 87.4(1) |
|  |  |  | O1*^a^* | Zn01 | N7 | 106.1(1) |
|  |  |  | O1*^a^* | Zn01 | N5 | 105.4(1) |
|  |  |  | O1*^a^* | Zn01 | N3 | 98.3(1) |
|  |  |  | O1*^a^* | Zn01 | N1 | 99.2(2) |
|  |  |  | N1 | Zn01 | N5 | 155.3(2) |
|  |  |  | N3 | Zn01 | N7 | 155.6(2) |

*^a^* O1 = aqua oxygen (H1A,H1B)

**Table S6.** Deviations of benzo ring centroids (yellow) and maximum atomic displacements (blue, Å) from the inner ring 16-atom macrocyclic plane (grey) for **ZnPc-2· A** (left) and **B** (right). Thermal ellipsoid model at 50% probability level. Solvent molecules, peripheral 2,6-dimethylphenyloxy substituents, and hydrogens omitted for clarity.


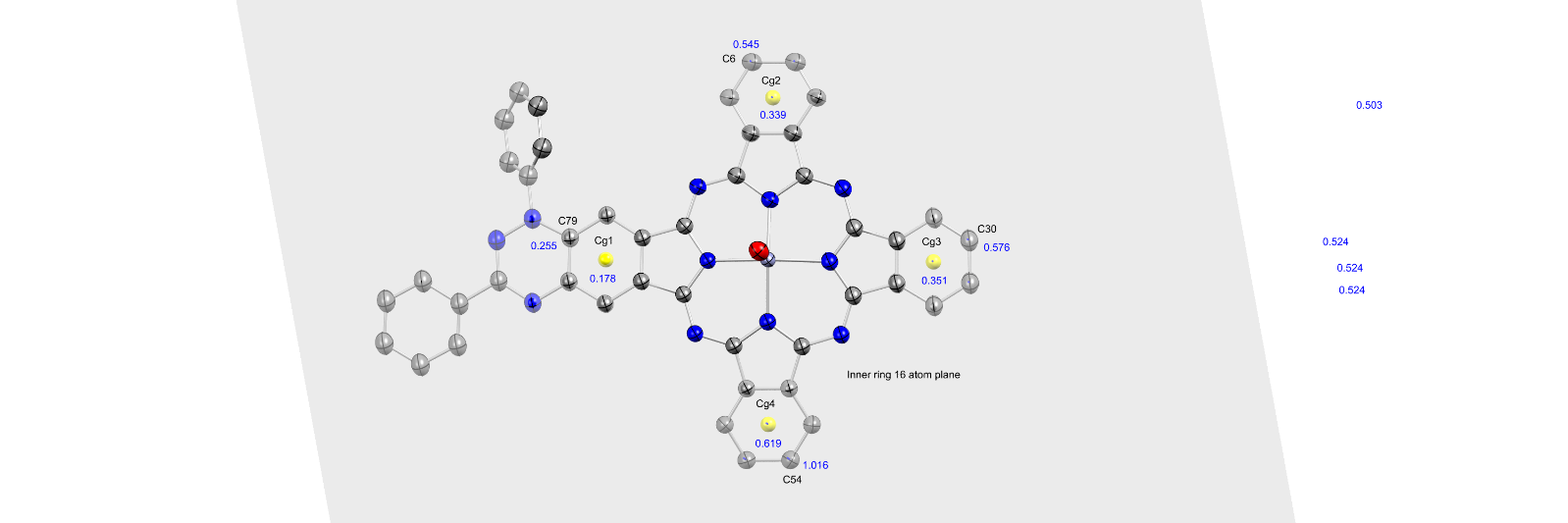

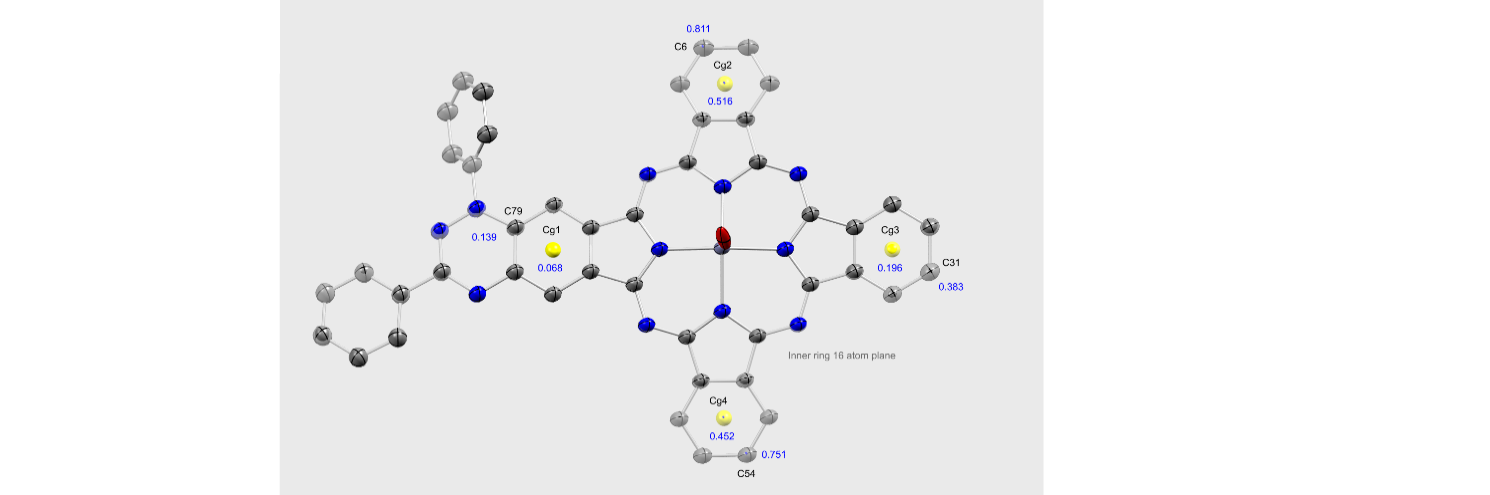


| **Benzo Ring (Atoms)** | **Centroid** | **Centroid Deviation from 16-Atom Plane**  **(Å)*^a^*** | **Ring Atom with Max Deviation from Plane**  **(Å)** |
| --- | --- | --- | --- |
| **ZnPc-2· A** |  |  |  |
| C77-C78-C79-C80-C75-C74 | Cg^1^ | 0.178 | C79, 0.255 |
| C2-C3-C5-C6-C7-C8 | Cg^2^ | 0.339 | C6, 0.545 |
| C32-C27-C26-C29-C30-C31 | Cg^3^ | 0.351 | C30, 0.576 |
| C55-C56-C51-C50-C53-C54 | Cg^4^ | 0.619 | C54, 1.016 |
| **ZnPc-2· B** |  |  |  |
| C77-C78-C79-C80-C75-C74 | Cg^1^ | 0.068 | C79, 0.139 |
| C2-C3-C5-C6-C7-C8 | Cg^2^ | 0.516 | C6, 0.811 |
| C32-C27-C26-C29-C30-C31 | Cg^3^ | 0.196 | C31, 0.383 |
| C55-C56-C51-C50-C53-C54 | Cg^4^ | 0.452 | C54, 0.751 |

*^a^* Inner 16-atom plane defined by N1-C1-N2-C25-N3-C28-N4-C49-N5-C52-N6-C73-N7-C76-N8-C4.

**Table S7.** Deviations of individual atoms from the 13-atom mean plane of the [1,2,4]triazino[5,6-*f*]isoindole unit in **ZnPc-2·** radicals **A** and **B**. ORTEP views of **ZnPc-2·** radicals **A** (left) And **B** (right) with deviations (blue, Å) indicated. Thermal ellipsoids are drawn at the 50% probability level; hydrogen atoms and co-crystallized solvent molecules omitted for clarity.


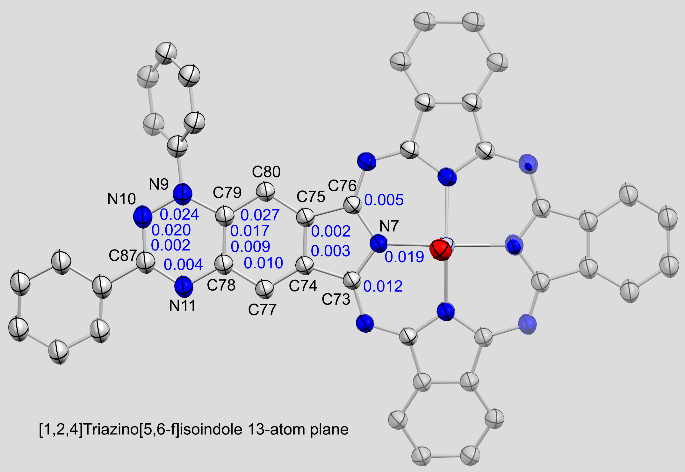

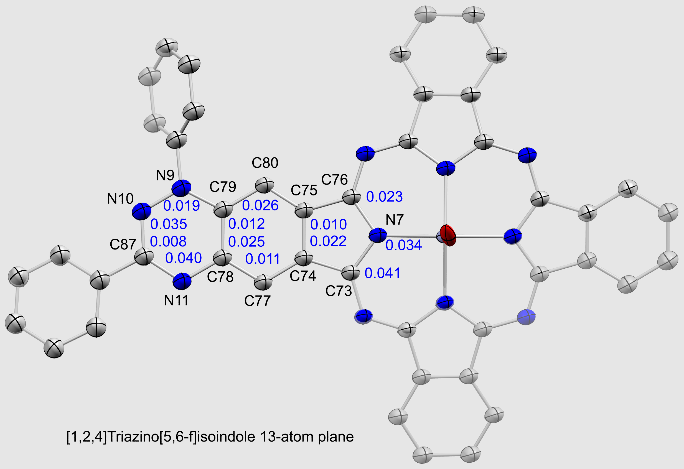


| **ZnPc-2· A** | | **ZnPc-2· B** | |
| --- | --- | --- | --- |
| **Atom**  **(#)** | **Deviation from 13-atom plane *^a^***  **(Å)** | **Atom**  **(#)** | **Deviation from 13-atom plane *^a^***  **(Å)** |
| N7 | 0.019 | N7 | 0.034 |
| C73 | 0.012 | C73 | 0.041 |
| C74 | 0.003 | C74 | 0.022 |
| C77 | 0.010 | C77 | 0.011 |
| C78 | 0.009 | C78 | 0.025 |
| N11 | 0.004 | N11 | 0.040 |
| C87 | 0.002 | C87 | 0.008 |
| N10 | 0.020 | N10 | 0.035 |
| N9 | 0.024 | N9 | 0.019 |
| C79 | 0.017 | C79 | 0.012 |
| C80 | 0.027 | C80 | 0.026 |
| C75 | 0.002 | C75 | 0.010 |
| C76 | 0.005 | C76 | 0.023 |

*^a^* 13-Atom plane of [1,2,4]triazino[5,6-*f*]isoindole unit defined by: N7-C73-C74-C66-C78-N11-C87-N10-N9-C79-C80-C75-C76.

Distortion-mode analysis using the extended *PorphyStruct*^9^ basis reveals that doming is the dominant non-planar deformation in both radicals (Fig. S18). **ZnPc-2A** displays a total out-of-plane displacement of *D*_oop_ = 0.495 Å, with doming as the primary component and smaller contributions from saddling and ruffling. **ZnPc-2B** exhibits a slightly larger distortion (*D*_oop_ = 0.539 Å), characterized by an enhanced saddling contribution, ruffling of comparable magnitude to **A**, and reduced doming. Waving (X/Y) and propelling modes are negligible in both structures. These quantitative mode profiles corroborate the geometric analysis and show that **ZnPc-2** radicals **A** and **B** adopt related but distinct asymmetrically domed conformations, reflecting subtle differences in the balance of doming, saddling, and ruffling influenced by the fused TAI unit.

**ZnPc-2· A**

**
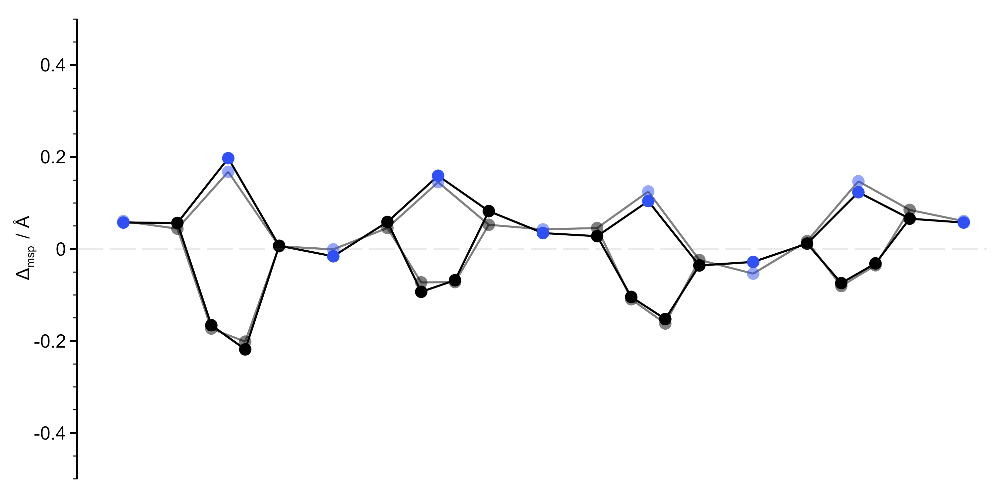

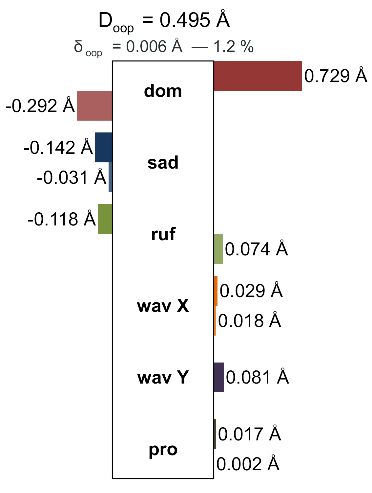
**

**ZnPc-2· B**


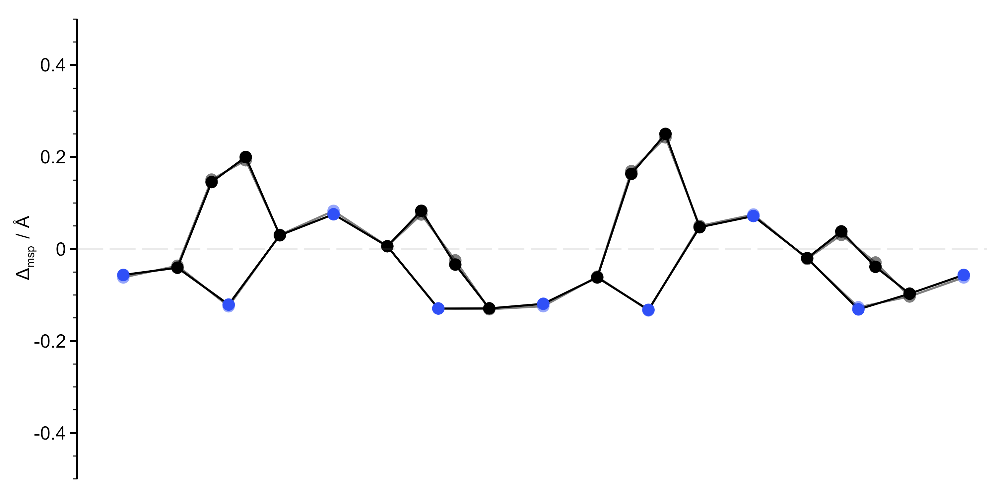

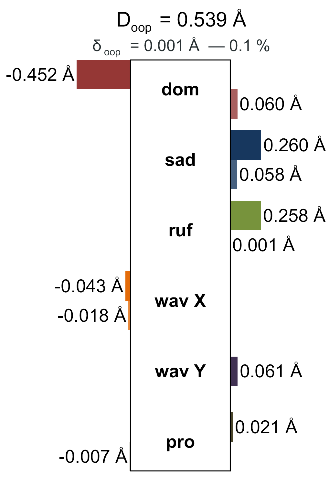


**Figure S18.** *PorphyStruct***^9^** results for **ZnPc-2·** radicals **A** (above) and **B** (below), using the extended basis. Left: Linear displacement diagram of non-planar distortions and simulated result overlapping. Right: Colour-coded bar plots of the contributions of the six lowest-energy porphyrin-like normal modes to the overall distortion *D*_oop_.

**ZnPc-2· A**

**
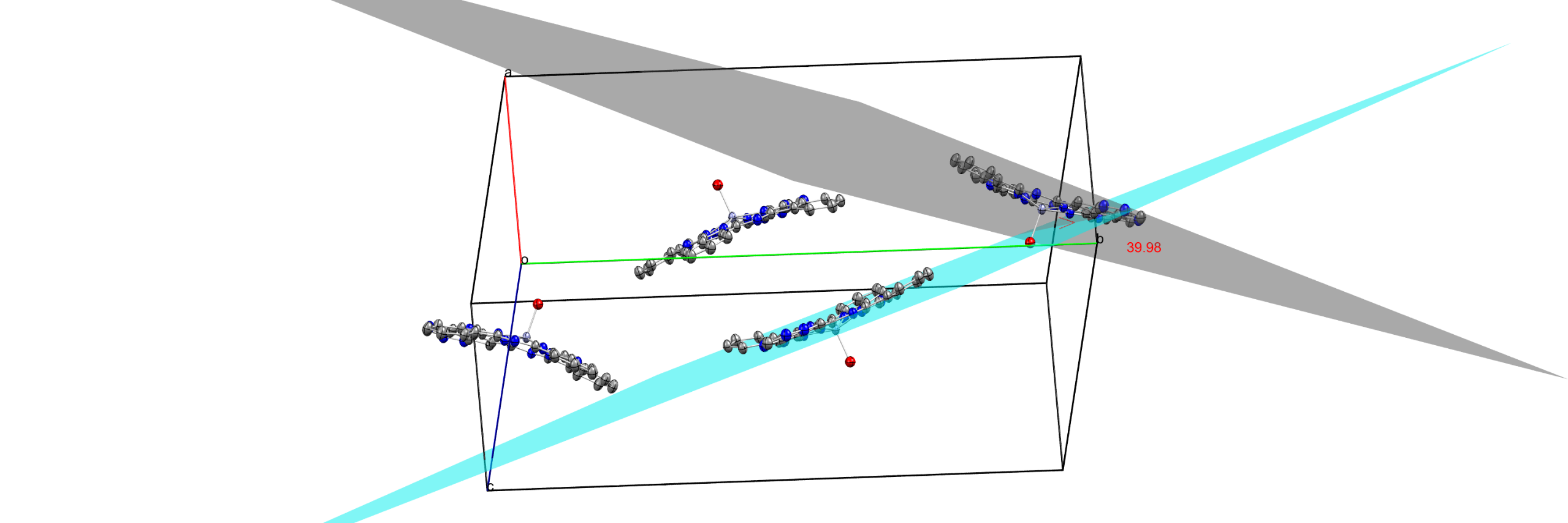
**

**ZnPc-2· B**

**
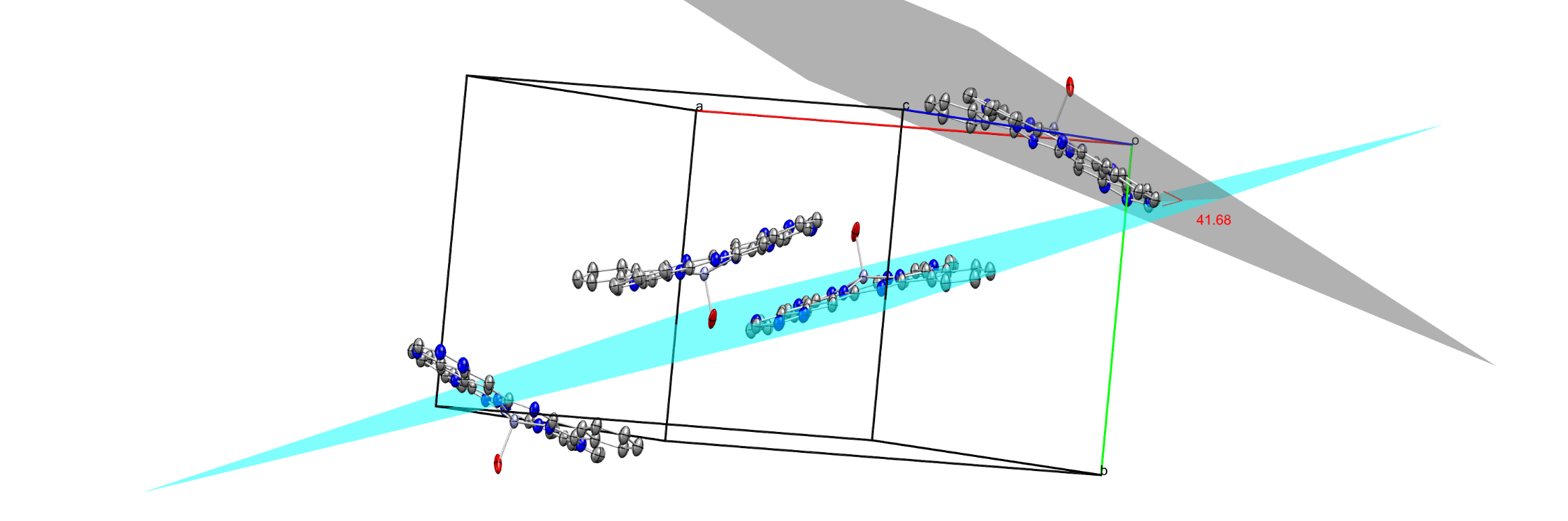
**

**Figure S19.** Relative orientations of adjacent **ZnPc-2·** molecules **A** (above) and **B** (below) within the herringbone motif and unit cell. The angles between the mean inner ring 16-atom planes (gray and light blue planes) of neighboring columns are 39.98° and 41.68° respectively. ORTEP representations (50% thermal ellipsoids) are used; solvent molecules, hydrogen atoms, and peripheral bis(1,6-dimethylphenoxy) and phenyl substituents are omitted for clarity.

**ZnPc-2· A**

**
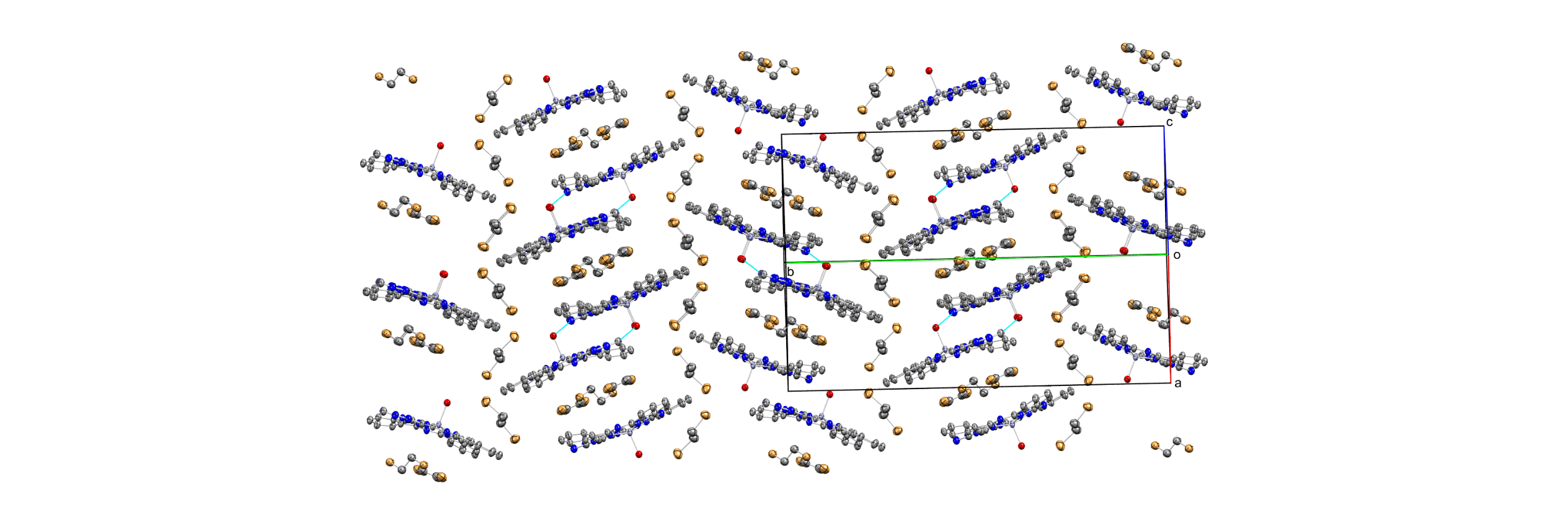
**

**ZnPc-2· B**

**
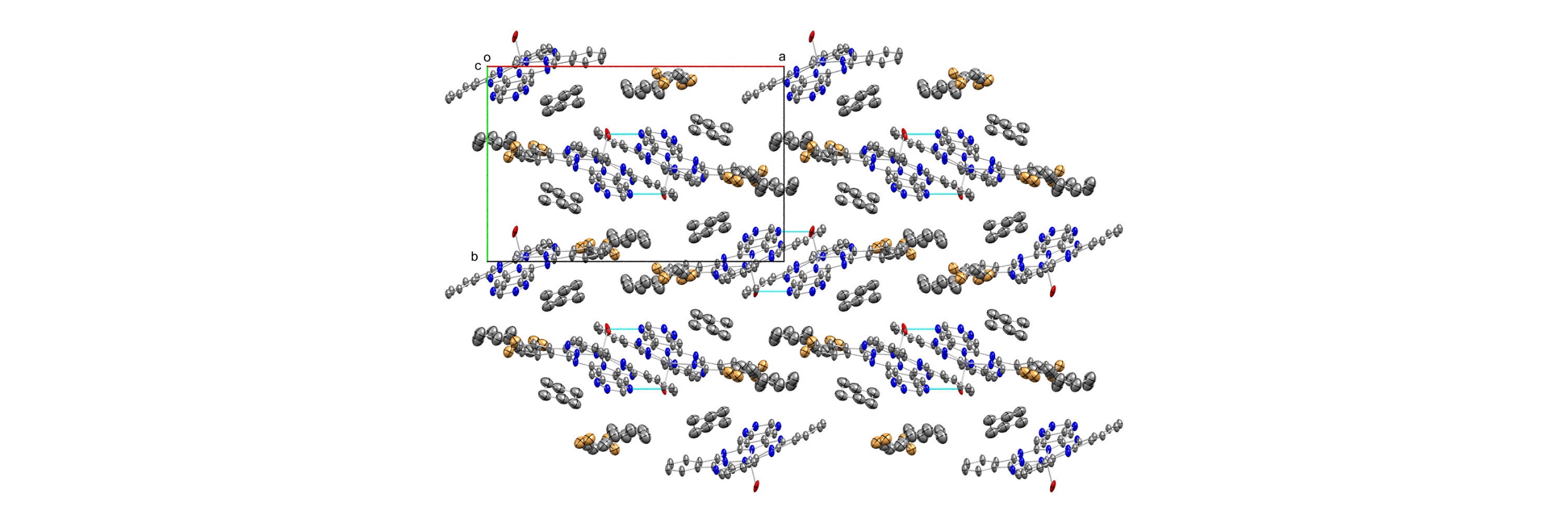
**

**Figure S20.** Crystal packing of **ZnPc-2·** molecules **A** (above) and **B** (below) in a 2×2×2 unit cell expansion, illustrating the herringbone arrangement. The original unit cell is outlined. Key hydrogen bonds are shown as dashed light-blue lines. ORTEP representations (50% thermal ellipsoids) are used; hydrogen atoms and peripheral bis(1,6-dimethylphenoxy) and phenyl substituents are omitted for clarity.

**Table S8.** *Cis*-oriented torsion angles (*Φ*, °) of the triazinyl–phenyl substituents in **ZnPc-2·** radicals **A** (left) and **B** (right). Only torsions involving atoms across the sigma bond connecting the triazinyl and phenyl groups are shown. ORTEP representations are shown with thermal ellipsoids at the 50% probability level. Solvent molecules, hydrogen atoms, and peripheral bis(1,6-dimethylphenoxy) substituents have been omitted for clarity.


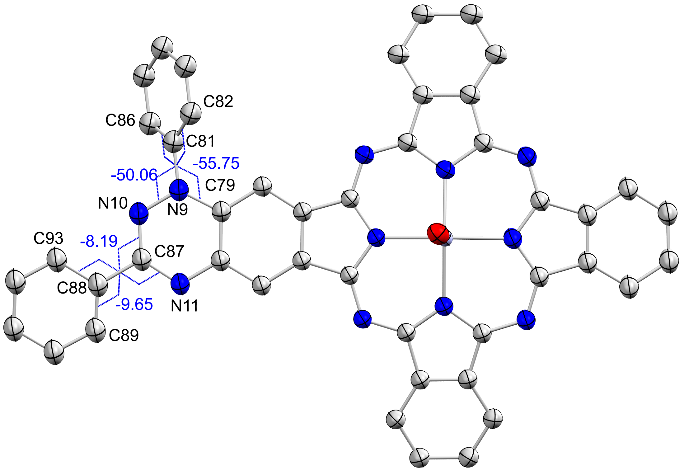

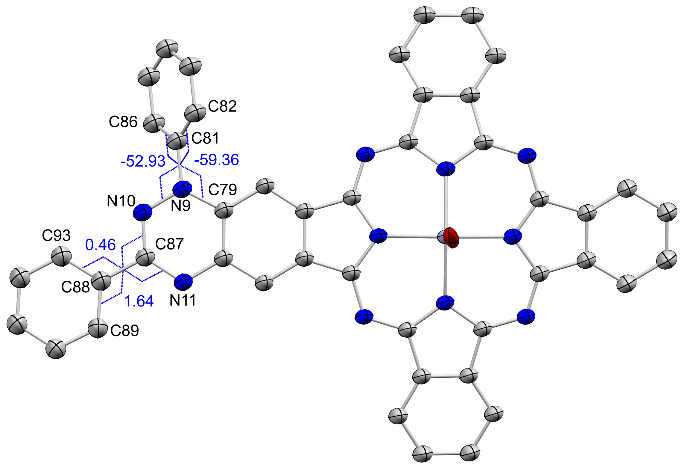


| **ZnPc-2·** | **N9-Ph** | | **C87-Ph** | |
| --- | --- | --- | --- | --- |
|  | **Torsion (*Φ*)**  **(Atoms)** | **Torsion (*Φ*)**  **(°)** | **Torsion (*Φ*)**  **(Atoms)** | **Torsion (*Φ*)**  **(°)** |
| **A** | C82-C81-N9-C79 | -55.75 | Ν11-C87-C88-C89 | -9.65 |
|  | C86-C81-N9-N10 | -50.06 | N10-C87-C88-C93 | -8.19 |
| **B** | C82-C81-N9-C79 | -59.36 | Ν11-C87-C88-C89 | 1.64 |
|  | C86-C81-N9-N10 | -52.93 | N10-C87-C88-C93 | 0.46 |

## Secondary and solvent interactions

Secondary interactions between peripheral bis(1,6-dimethylphenoxy) substituents propagate the herringbone motifs (Table S7). In **ZnPc-2· A**, inversion-related dimers interact via a nearly linear C–H···*π* contact between C11 and the centroid of phenyl ring C33–C38 (Cg^1^) (C11–H11···Cg^1^ = 3.662 Å, H11···Cg^1^ = 2.720 Å and ∠C11–H11···Cg^1^ = 171.93°), complemented by a C–H···O hydrogen bond (C39–H39A···O7 = 3.404 Å, H39A···O7 = 2.550 Å, ∠C39–H39···O7 = 145.31°). In **ZnPc-2· B**, a C–H···*π* interaction occurs between C39 and the pyrrole ring centroid C1–C4/N1 (Cg^2^) (C39–H39A···Cg^2^ = 3.953 Å, H39A···Cg^2^ = 2.984 Å and ∠C39–H39A···Cg^2^ = 170.70°), along with a C–H···metallacycle contact involving C35 and the Zn-cantered macrocyclic ring C4 N8 C76 N7 Zn01–N1 (Cg^3^) (C35–H35···Cg^3^ = 3.306 Å, H35···Cg^3^ = 2.468 Å and ∠C35–H35···Cg^3^ = 147.00°).

Solvent molecules further contribute to weak contacts (Table S7). In **ZnPc-2· A** (three DBE per unit cell), a bent O–H···Br contact is observed [O1–H1B···Br = 3.471 Å, H1B···Br4 = 2.929 Å, ∠O1–H1B···Br4 = 115.72°], along with C–H···N and C–H···Br interactions [C–H ···N = 3.34–3.68 Å; H···N = 2.50–2.74 Å; ∠C–H···N = 142–159°; C64–H64C···Br5 = 3.590 Å, H64C···Br5 = 2.740 Å, ∠C64–H64C···Br5 = 145.38°], and weaker C–H···*π* interactions. In **ZnPc-2· B** (two cyclohexane and one partially disordered DBE), a C–H···N contact [C97–H97A···N2 = 3.530 Å, H97A···N2 = 2.617 Å, ∠C97–H97A···N2 = 153.34°] and weaker C–H···*π* [C98–H98A···Cg^3^ = 3.606 Å, H98A···Cg^3^ = 2.673 Å, ∠C98–H98A···Cg^3^ = 157.04°] and C–H···C(aryl) contacts are observed.

**Table S9.** Solvent and secondary interactions within phthalocyanine radicals **ZnPc-2· A** and **B**.

| **D–H···A** | **Centroid Atoms**  **(Cg^n^)** | **D···A**  **(Å)** | **H···A**  **(Å)** | **∠D–H···A**  **(°)** | **Interaction** |
| --- | --- | --- | --- | --- | --- |
| **ZnPc-2· A** |  |  |  |  |  |
| C11–H11···Cg^1^ | Cg^1^: C33-C38 | 3.662 | 2.720 | 171.93 | Inter Dimer C–H···*π* |
| C39–H39A···O7 |  | 3.404 | 2.550 | 145.31 | C–H···O hydrogen bond |
| O1–H1B···Br4 |  | 3.471 | 2.929 | 115.72 | Solvent O–H···Br |
| C96–H96A···N2 |  | 3.337 | 2.498 | 142.33 | Secondary contact |
| C94–H94B···N4 |  | 3.675 | 2.735 | 158.63 | Secondary contact |
| C64–H64C···Br5 |  | 3.590 | 2.740 | 145.38 | Secondary contact |
| C98–H98A···Cg^2^ | Cg^2^: C73 N7 Zn01 N5 C52 N6 | 3.217 | 2.646 | 116.93 | C–H···metallacycle |
| C95–H95B···Cg^3^ | Cg^3^: C29 C26 C27 C32 C31 C30 | 3.672 | 2.740 | 156.92 | C–H···*π* |
| **ZnPc-2· B** |  |  |  |  |  |
| C39–H39A···Cg^1^ | Cg^1^: C1–C4/N1 | 3.953 | 2.984 | 170.70 | C–H···*π* |
| C35–H35···Cg^2^ | Cg^2^: C4 N8 C76 N7 Zn01–N1 | 3.306 | 2.468 | 147.00 | C–H···metallacycle |
| C98–H98A···Cg^3^ | Cg^3^: C25 N3 C28 C27 C26 | 3.606 | 2.673 | 157.04 | C–H···*π* |
| C97–H97A···N2 |  | 3.530 | 2.617 | 153.34 | C–H···N |
| C99–H99B···C1 |  | 3.755 | 2.798 | 162.63 | C–H···C(aryl, quaternary) |
| C101–H10F···C78 |  | 3.846 | 2.887 | 163.45 | C–H···C(aryl, quaternary) |

# Computational Studies

## Computational methodology

All calculations were performed using the *Gaussian 16* (Revision A.03) suite of programs.**^10^** Single-point (SP) and time-dependent (TD) calculations were conducted on the X-ray crystal structures of **ZnPc-2· A** and **B** radicals, with co-crystallized solvent molecules removed, using unrestricted density functional theory (UDFT) with the B3LYP hybrid exchange–correlation functional and the 6-31G(2d,p) basis set.**^11^** For the TD-DFT calculations, 45 excited states were computed to describe the low-energy region of the UV–vis absorption spectra.

The LUMO and LUMO+1 energies were determined from the SOMOα energies obtained in the SP calculations and the corresponding excited-state energies from the TD-DFT results, according to Equations 1 and 2.

**Equation 1**

$$\boldsymbol{E}_{\mathbf{LUMO}}\boldsymbol{=}\boldsymbol{E}_{\mathbf{SOMO}\alpha}\boldsymbol{+}\boldsymbol{E}_{\mathbf{SOMO}\alpha\boldsymbol{\to LUMO}\alpha}$$

**Equation 2**

$$\boldsymbol{E}_{\mathbf{LUMO+1}}\boldsymbol{=}\boldsymbol{E}_{\mathbf{SOMO}\alpha}\boldsymbol{+}\boldsymbol{E}_{\mathbf{SOMO}\alpha\boldsymbol{\to LUMO+1}\alpha}$$

The predicted UV-vis spectra for **ZnPc-2· A** and **B** were generated from the allowed excited states (2*S*+1 < 2.5-A and ⟨*S*²⟩ < 1.3) computed at the TD-DFT/UB3LYP 6-31G(2d,p) level, using equation 3**^12^** with a Gaussian broadening factor (σ) of 3226 cm^−1^.

**Equation 3**

$$\epsilon=\sum_{i=1}^{n} \mathrm{Factor}\times\frac{f_{i}}{\sigma}\times\exp\left( -2.772\times\left( \frac{\lambda_{\mathrm{plot}}-\lambda_{i}}{\sigma} \right)^{2} \right)$$

## Further discussion

Single point calculations on both **ZnPc-2· A** and **B** revealed minor differences in the electronic energies of the two X-ray structures: the ground state energy has only 0.236 Hartree difference and the frontier molecular orbitals (FMOs) differ from 0.006 (SOMOα) to 0.04 (LUMO+1α) eV (Table S10). These minor differences can be attributed to the slight deviations between the two structures and mainly the difference in the axial Zn-OH_2_ distance. Thus, the **ZnPc-2· B** calculations, being in an overall lower thermodynamically energetic state is discussed in the main manuscript.

TD-DFT calculations for both **ZnPc-2· A** and **B** give similar UV-vis spectra (Fig. S19); a result of similarities in the theoretical excited states (Tables S11 & S12), thus only **ZnPc-2· B** is discussed extensively in the main manuscript.

**Table S10.** Computational results for **ZnPc-2· A** and **B** radicals as calculated at the DFT UB3LYP/6-31G(2d,p) level of theory.

| **DFT UB3LYP 6-31G(2d,p)** | ***E*_GS_**  **(Hartree)** | **Dipole**  **(D)** | ***E*_HOMO_**_−_**_1_**  **(eV)** | ***E*_HOMOα_**  **(eV)** | ***E*_HOMOβ_**  **(eV)** | ***E*_SOMOα_**  **(eV)** | ***E*_LUMOα_**  **(eV)** | ***E*_LUMO+1α_**  **(eV)** |
| --- | --- | --- | --- | --- | --- | --- | --- | --- |
| **ZnPc-2· A** | −6495.587 | 4.452 | −5.545 | −4.494 | −4.445 | −4.311 | −2.943 | −2.648 |
| **ZnPc-2· B** | −6495.823 | 3.646 | −5.558 | −4.480 | −4.439 | −4.349 | −2.967 | −2.688 |
| \|Diff\| | 0.236 | 0.806 | 0.013 | 0.014 | 0.006 | 0.038 | 0.024 | 0.040 |

**Table S11.** Selected TD-DFT excited states of **ZnPc-2· A** [UB3LYP/6-31G(2d,p)]. Radical-specific SOMO excitations are highlighted; states with minor quartet contamination are labelled as partially allowed.

| **Excited**  **State** | **Energy**  **(eV)** | ***λ***  **(nm)** | ***f*** | **<*S*²>** | **2*S*+1** | **Dominant transitions** | **Radical-specific?** | **Allowed** |
| --- | --- | --- | --- | --- | --- | --- | --- | --- |
| 3 | 1.368 | 906.5 | 0.0123 | 0.860 | 2 | SOMOα → LUMOα | Yes | Partially |
| 4 | 1.615 | 767.8 | 0.0391 | 1.125 | 2 | HOMOβ → SOMOβ | Yes | Partially |
| 5 | 1.664 | 745.3 | 0.0210 | 0.811 | 2 | SOMOα → LUMO+1α | Yes | Yes |
| 6 | 1.999 | 620.1 | 0.6913 | 0.777 | 2 | HOMOα,β → LUMOα,β | No | Yes |
| 7 | 2.090 | 593.2 | 0.3929 | 0.780 | 2 | HOMOα,β → LUMO+1α,β | No | Yes |
| 19 | 2.797 | 443.2 | 0.0500 | 1.256 | 2 | HOMO–1α,β → LUMOα,β | No | Partially |
| 28 | 2.999 | 413.5 | 0.0994 | 1.100 | 2 | HOMO–3α → LUMO+1α | No | Partially |
| 30 | 3.0386 | 408.0 | 0.0432 | 1.074 | 2 | HOMO–2β → LUMO+1β | No | Partially |
| 40 | 3.2215 | 384.9 | 0.0442 | 1.029 | 2 | HOMOα,β → LUMO+2α,β | No | Partially |

**Table S12.** Selected TD-DFT excited states of **ZnPc-2· B** [UB3LYP/6-31G(2d,p)]. Radical-specific SOMO excitations are highlighted; states with minor quartet contamination are labelled as partially allowed.

| **Excited**  **State** | **Energy**  **(eV)** | **λ**  **(nm)** | **f** | **<S²>** | **2S+1** | **Dominant**  **Orbitals** | **Radical-specific?** | **Allowed** |
| --- | --- | --- | --- | --- | --- | --- | --- | --- |
| 3 | 2.099 | 897 | 0.0018 | 0.852 | 2 | SOMOα → LUMOα | Yes | Partially |
| 4 | 2.322 | 776 | 0.0307 | 1.098 | 2 | HOMOβ → SOMOβ | Yes | Partially |
| 5 | 2.055 | 746 | 0.0126 | 0.806 | 2 | SOMOα → LUMO+1α | Yes | Yes |
| 6 | 2.021 | 627 | 0.7075 | 0.771 | 2 | HOMOα,β → LUMOα,β | No | Yes |
| 7 | 2.031 | 598 | 0.3910 | 0.782 | 2 | HOMOα,β → LUMO+1α,β | No | Yes |
| 21 | 2.190 | 436 | 0.0618 | 0.949 | 2 | HOMO–1/–2/–3 → LUMO/LUMO+1 | No | Yes |
| 23 | 2.170 | 432 | 0.2107 | 0.927 | 2 | HOMO–1/–2/–3 → LUMO/LUMO+1 | No | Yes |
| 32 | 3.102 | 400 | 0.107 | 1.003 | 2 | HOMO–1/–2/–3β → LUMO+1β | No | Partially |

**Figure S21.** Simulated UV-vis spectrum of **ZnPc-2·** molecules **A** (red) and **B** (blue) as plotted from the excited states calculated at TD-DFT/UB3LYP 6-31G(2d,p) level of theory, with σ = 3226 cm^−1^.

**
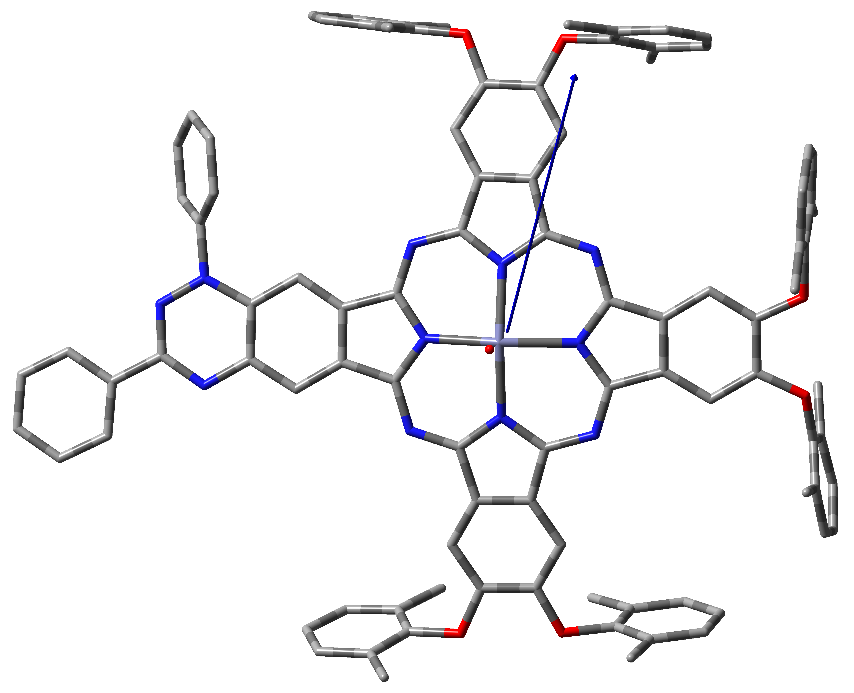
**

**Figure S22.** Dipole vector of **ZnPc-2· B** as calculated at TD-DFT/UB3LYP 6-31G(2d,p) level of theory.

.


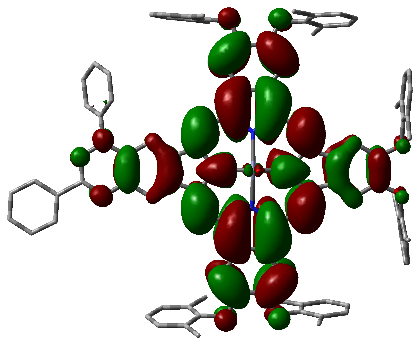
*E*_LUMO+1_ = −2.688 eV


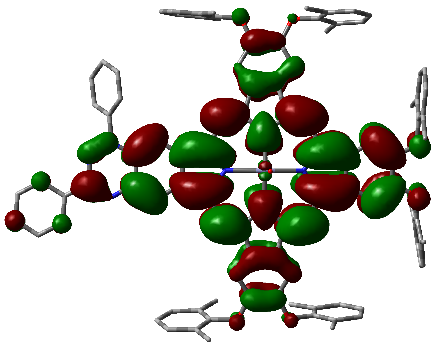
*E*_LUMO_ = −2.967 eV


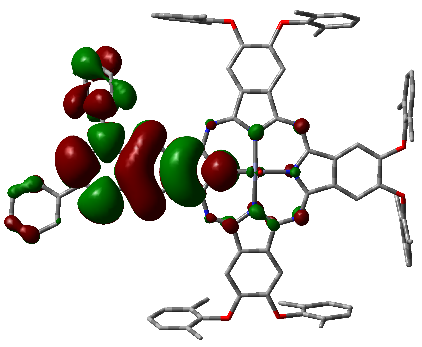
*E*_SOMO_ = −4.349 eV


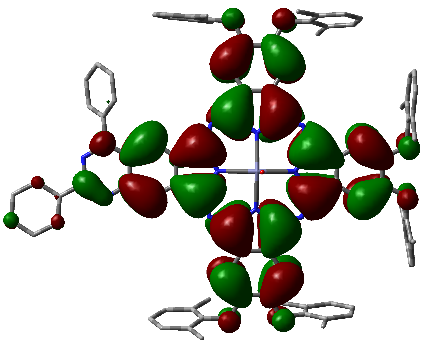
*E*_HOMO_ = −4.480 eV


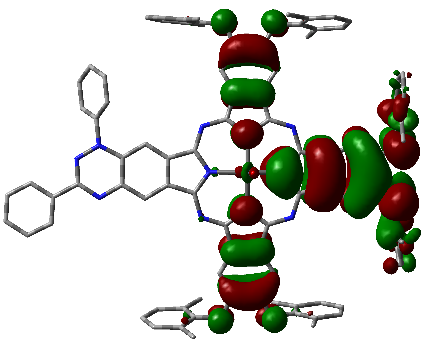
*E*_HOMO−1_ = −5.558 eV

**Figure S23.** Frontier molecular orbital (FMOs) surfaces for **ZnPc-2· B** radical as calculated at the DFT UB3LYP/6-31G(2d,p) level of theory.


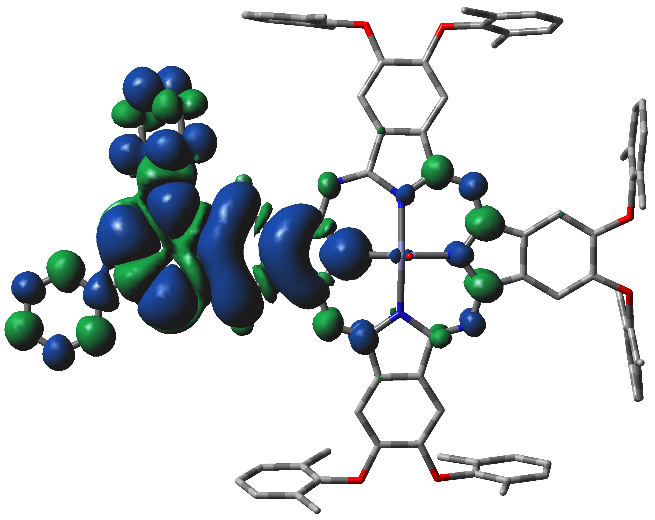


**Figure S24.** Spin density map of **ZnPc-2· B** as calculated with UB3LYP/6-31G(2d,p). The α and β spin densities are rendered in blue and green, respectively, using an MO and density isovalues of 0.0002 a.u. The visualization highlights the spatial distribution of unpaired electrons within the molecular framework, emphasizing regions of net spin polarization, as well as the minimal effect of the ligand to the spin distribution.


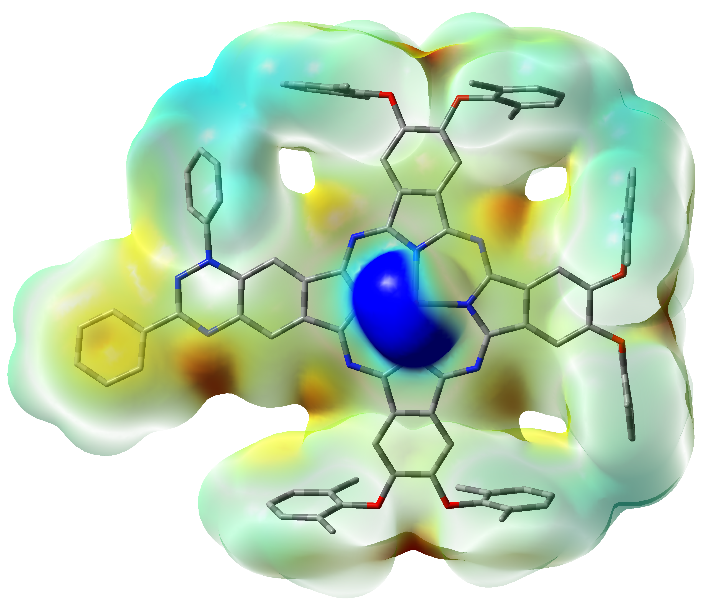


**Figure** **S25.** Molecular electrostatic potential (ESP) surface of **ZnPc-2· B** as calculated with UB3LYP/6-31G(2d,p). The ESP is mapped onto the electron density isosurface, red color indicating an electron-rich regions and blue color indicating electron-deficient regions. Green color denotes regions of near-zero potential, providing a visual reference for electrostatic neutrality across the molecular surface

## Output data from TD-DFT calculations at UB3LYP/6-31G(2d,p) (45 excited states)

**ZnPc-2· A**

Excited State 1: 3.389-A 1.0820 eV 1145.91 nm f=0.0012 <S**2>=2.621

397A -> 399A 0.60980

397A -> 400A -0.15215

398A -> 399A -0.26158

397B -> 398B 0.54205

397B -> 399B 0.45745

397B -> 400B -0.19430

397A <- 399A 0.11051

This state for optimization and/or second-order correction.

Total Energy, E(TD-HF/TD-DFT) = -6495.58720176

Copying the excited state density for this state as the 1-particle RhoCI density.

Excited State 2: 3.331-A 1.1785 eV 1052.01 nm f=0.0016 <S**2>=2.523

397A -> 399A 0.20410

397A -> 400A 0.56735

398A -> 399A -0.12840

398A -> 400A -0.24086

397B -> 398B -0.43409

397B -> 399B 0.52334

397B -> 400B 0.31899

Excited State 3: 2.107-A 1.3677 eV 906.51 nm f=0.0123 <S**2>=0.860

397A -> 399A 0.26443

397A -> 400A 0.15186

398A -> 399A 0.89866

398A -> 401A 0.14468

397B -> 398B 0.20732

397B -> 400B 0.14156

Excited State 4: 2.345-A 1.6149 eV 767.76 nm f=0.0391 <S**2>=1.125

397A -> 399A -0.25952

397A -> 400A 0.36428

398A -> 399A -0.20555

397B -> 398B 0.62000

397B -> 399B -0.15367

397B -> 400B 0.56812

Excited State 5: 2.060-A 1.6635 eV 745.33 nm f=0.0210 <S**2>=0.811

397A -> 400A 0.20354

398A -> 400A 0.94426

397B -> 398B -0.10644

397B -> 399B 0.18455

Excited State 6: 2.024-A 1.9994 eV 620.10 nm f=0.6913 <S**2>=0.774

397A -> 399A 0.64455

397A -> 400A 0.14533

398A -> 399A -0.17113

398A -> 400A 0.14526

397B -> 398B -0.18408

397B -> 399B -0.63642

397B -> 400B 0.16480

Excited State 7: 2.029-A 2.0900 eV 593.22 nm f=0.3929 <S**2>=0.780

397A -> 400A -0.63572

398A -> 400A 0.11436

397B -> 398B -0.16718

397B -> 399B 0.16841

397B -> 400B 0.67234

Excited State 8: 3.244-A 2.4077 eV 514.96 nm f=0.0002 <S**2>=2.380

396A -> 399A 0.18153

396A -> 400A -0.50641

398A -> 401A -0.14557

396B -> 398B 0.65721

396B -> 399B -0.15260

396B -> 400B -0.39033

Excited State 9: 2.134-A 2.4250 eV 511.28 nm f=0.0090 <S**2>=0.889

396A -> 400A -0.10023

397A -> 401A 0.12325

398A -> 399A -0.12432

398A -> 401A 0.74026

398A -> 402A 0.10307

398A -> 405A -0.12093

398A -> 407A 0.15160

392B -> 398B 0.40208

392B -> 400B 0.17109

393B -> 398B 0.22045

396B -> 398B 0.11220

Excited State 10: 3.313-A 2.4993 eV 496.08 nm f=0.0008 <S**2>=2.493

395A -> 400A -0.56619

395B -> 398B 0.61108

395B -> 399B -0.19240

395B -> 400B -0.41334

395B -> 401B 0.10982

Excited State 11: 3.449-A 2.5302 eV 490.01 nm f=0.0003 <S**2>=2.723

394A -> 399A 0.59228

395A -> 399A -0.19905

396A -> 399A -0.24849

394B -> 398B 0.30534

394B -> 399B 0.48337

394B -> 401B 0.10583

395B -> 398B -0.11918

395B -> 399B -0.16307

396B -> 399B -0.21692

Excited State 12: 3.446-A 2.6102 eV 475.00 nm f=0.0006 <S**2>=2.719

395A -> 399A -0.35763

395A -> 400A -0.17930

396A -> 399A 0.56825

396A -> 400A 0.19681

395B -> 398B -0.20428

395B -> 399B -0.33431

396B -> 398B 0.11255

396B -> 399B 0.49200

Excited State 13: 3.341-A 2.6440 eV 468.92 nm f=0.0010 <S**2>=2.540

394A -> 399A 0.29550

395A -> 399A 0.41740

395A -> 400A 0.25364

396A -> 399A 0.30965

396A -> 400A 0.12101

394B -> 398B 0.17462

394B -> 399B 0.23659

395B -> 398B 0.39908

395B -> 399B 0.38614

396B -> 398B 0.13828

396B -> 399B 0.29682

Excited State 14: 2.094-A 2.6691 eV 464.52 nm f=0.0048 <S**2>=0.847

398A -> 401A 0.29801

374B -> 398B 0.16597

374B -> 400B 0.10113

377B -> 398B 0.10410

378B -> 398B 0.58840

378B -> 399B -0.17816

378B -> 400B 0.34446

379B -> 398B 0.11707

382B -> 398B -0.17908

382B -> 400B -0.10148

392B -> 398B -0.35357

392B -> 400B -0.11021

393B -> 398B -0.19137

Excited State 15: 2.203-A 2.6882 eV 461.22 nm f=0.0316 <S**2>=0.963

395A -> 400A -0.11528

396A -> 399A -0.40399

396A -> 400A 0.61007

396B -> 398B 0.62393

Excited State 16: 2.351-A 2.7504 eV 450.79 nm f=0.0152 <S**2>=1.132

394A -> 399A -0.14394

394A -> 400A -0.14278

395A -> 399A 0.52586

395A -> 400A -0.37798

396A -> 399A 0.15620

378B -> 398B -0.13073

392B -> 398B -0.13323

394B -> 398B 0.34386

394B -> 400B -0.13650

395B -> 398B -0.48304

396B -> 399B -0.13373

Excited State 17: 2.235-A 2.7614 eV 449.00 nm f=0.0073 <S**2>=0.998

395A -> 399A 0.20793

395A -> 400A -0.13807

398A -> 401A -0.33529

398A -> 402A 0.35197

398A -> 403A 0.10046

398A -> 404A -0.12115

374B -> 398B 0.11453

378B -> 398B 0.38915

378B -> 399B -0.11138

378B -> 400B 0.21059

392B -> 398B 0.46891

393B -> 398B 0.25586

Excited State 18: 2.575-A 2.7836 eV 445.41 nm f=0.0333 <S**2>=1.407

394A -> 399A -0.27611

394A -> 400A -0.27126

395A -> 399A -0.25859

395A -> 400A 0.29787

396A -> 399A 0.15352

396A -> 400A 0.24396

398A -> 402A -0.12000

392B -> 398B 0.10147

394B -> 398B 0.55285

394B -> 400B -0.19292

395B -> 398B 0.23557

396B -> 399B -0.33691

Excited State 19: 2.454-A 2.7973 eV 443.23 nm f=0.0500 <S**2>=1.256

394A -> 400A -0.22684

396A -> 399A -0.43585

396A -> 400A -0.29958

394B -> 398B 0.36496

394B -> 399B -0.26523

394B -> 400B -0.19408

396B -> 399B 0.59381

Excited State 20: 2.402-A 2.8340 eV 437.48 nm f=0.0074 <S**2>=1.193

387A -> 399A 0.14820

394A -> 399A 0.18760

395A -> 399A -0.26774

395A -> 400A -0.27359

396A -> 399A 0.10285

397A -> 401A -0.16848

397A -> 402A -0.10573

398A -> 402A -0.38670

398A -> 404A 0.11767

385B -> 398B -0.16140

387B -> 398B 0.22231

387B -> 399B 0.11789

387B -> 400B -0.10782

392B -> 398B 0.10532

394B -> 398B -0.12410

394B -> 399B -0.21128

395B -> 399B 0.50293

395B -> 400B 0.10280

Excited State 21: 2.669-A 2.8470 eV 435.49 nm f=0.0260 <S**2>=1.531

385A -> 399A 0.13628

387A -> 399A -0.20720

394A -> 399A 0.17602

394A -> 400A -0.18436

395A -> 399A -0.29671

395A -> 400A -0.24818

397A -> 401A 0.21152

398A -> 402A 0.34305

398A -> 404A -0.10983

385B -> 398B 0.12187

387B -> 398B -0.20733

387B -> 399B -0.11197

394B -> 398B 0.15456

394B -> 399B -0.17714

394B -> 400B -0.10357

395B -> 399B 0.44091

397B -> 401B -0.17332

Excited State 22: 3.236-A 2.8582 eV 433.78 nm f=0.0055 <S**2>=2.368

384A -> 399A -0.20141

385A -> 399A -0.22401

387A -> 399A 0.38410

388A -> 399A -0.16335

397A -> 401A 0.29310

398A -> 402A 0.30937

384B -> 399B 0.12720

385B -> 398B -0.11747

385B -> 399B -0.18731

387B -> 398B 0.29880

387B -> 399B 0.32364

392B -> 398B -0.13531

392B -> 399B -0.10495

397B -> 401B -0.24223

Excited State 23: 3.452-A 2.8790 eV 430.65 nm f=0.0011 <S**2>=2.729

391A -> 403A 0.16621

393A -> 403A -0.60325

393A -> 404A -0.11943

393A -> 407A -0.18144

390B -> 402B -0.16286

392B -> 402B -0.26551

393B -> 402B 0.52601

393B -> 403B 0.17787

393B -> 407B 0.12542

393A <- 403A -0.11408

Excited State 24: 2.800-A 2.8853 eV 429.72 nm f=0.0418 <S**2>=1.710

393A -> 403A 0.11001

394A -> 399A 0.36573

394A -> 400A -0.21765

395A -> 399A 0.11083

395A -> 400A 0.14760

396A -> 399A 0.10613

397A -> 401A -0.31265

397A -> 402A 0.17137

398A -> 401A 0.14505

398A -> 402A 0.25852

398A -> 404A -0.10590

392B -> 399B 0.12887

394B -> 398B -0.10436

394B -> 399B -0.37287

395B -> 399B -0.15092

396B -> 399B -0.13605

397B -> 401B 0.39501

Excited State 25: 2.658-A 2.8887 eV 429.21 nm f=0.1162 <S**2>=1.517

387A -> 399A -0.14405

394A -> 399A 0.41051

394A -> 400A -0.12853

395A -> 399A 0.22492

395A -> 400A 0.13890

397A -> 401A 0.28740

397A -> 402A -0.15166

398A -> 401A -0.17468

398A -> 402A -0.24181

392B -> 398B 0.11741

392B -> 399B -0.14371

394B -> 399B -0.35919

395B -> 399B -0.26832

397B -> 401B -0.35483

Excited State 26: 3.306-A 2.9420 eV 421.43 nm f=0.0067 <S**2>=2.483

384A -> 400A -0.24730

385A -> 400A -0.25901

387A -> 399A 0.15306

387A -> 400A 0.42168

388A -> 400A -0.13978

398A -> 402A -0.13374

384B -> 399B 0.14352

385B -> 399B -0.22305

385B -> 400B -0.14726

387B -> 398B -0.37873

387B -> 399B 0.36711

387B -> 400B 0.28276

388B -> 399B 0.11243

Excited State 27: 2.212-A 2.9843 eV 415.46 nm f=0.0072 <S**2>=0.973

394A -> 400A 0.36980

396A -> 400A -0.21174

394B -> 398B 0.17281

394B -> 399B -0.21505

396B -> 398B 0.26950

396B -> 399B -0.16265

396B -> 400B 0.76480

Excited State 28: 2.324-A 2.9986 eV 413.47 nm f=0.0994 <S**2>=1.100

394A -> 400A 0.66495

396A -> 400A 0.12284

385B -> 398B -0.12697

394B -> 398B 0.29863

394B -> 399B -0.35172

395B -> 400B 0.16556

396B -> 398B -0.14790

396B -> 400B -0.39406

Excited State 29: 3.068-A 3.0090 eV 412.04 nm f=0.0088 <S**2>=2.103

384A -> 399A 0.18295

384A -> 400A -0.13728

385A -> 399A 0.19922

385A -> 400A -0.15139

387A -> 399A 0.14634

380B -> 398B -0.10686

381B -> 398B 0.12504

384B -> 398B -0.32118

384B -> 400B 0.14409

385B -> 398B 0.49983

385B -> 399B 0.12238

385B -> 400B -0.23097

387B -> 398B 0.41004

387B -> 399B 0.14657

387B -> 400B -0.11747

388B -> 398B 0.20407

396B -> 400B -0.14062

Excited State 30: 2.301-A 3.0386 eV 408.03 nm f=0.0432 <S**2>=1.074

394A -> 400A -0.20463

395A -> 399A 0.10479

395A -> 400A -0.24713

392B -> 399B 0.17848

395B -> 398B 0.25548

395B -> 399B -0.24524

395B -> 400B 0.77728

Excited State 31: 3.142-A 3.0551 eV 405.82 nm f=0.0049 <S**2>=2.218

385A -> 399A -0.10291

388A -> 399A 0.14988

390A -> 399A -0.28907

397A -> 401A 0.16652

397A -> 402A -0.13130

392B -> 399B 0.55643

392B -> 400B -0.17322

393B -> 399B 0.31176

395B -> 399B 0.14294

395B -> 400B -0.27831

397B -> 401B -0.19797

397B -> 403B 0.11378

Excited State 32: 3.148-A 3.0782 eV 402.78 nm f=0.0070 <S**2>=2.228

381A -> 399A 0.10827

382A -> 399A 0.13110

384A -> 399A 0.34795

384A -> 400A 0.15169

385A -> 399A 0.33297

385A -> 400A 0.16268

387A -> 399A 0.46657

388A -> 399A -0.21251

384B -> 399B -0.18877

385B -> 398B -0.14835

385B -> 399B 0.31823

385B -> 400B 0.12817

387B -> 398B -0.18110

387B -> 399B 0.14830

392B -> 399B 0.13762

Excited State 33: 3.313-A 3.1139 eV 398.16 nm f=0.0221 <S**2>=2.495

384A -> 399A 0.12877

387A -> 399A 0.16450

390A -> 399A -0.10191

397A -> 401A 0.15309

397A -> 402A 0.44286

397A -> 404A 0.24789

397A -> 407A -0.11002

398A -> 402A -0.20410

382B -> 398B -0.10999

392B -> 398B 0.10459

392B -> 399B 0.15403

397B -> 402B 0.14527

397B -> 403B -0.41297

397B -> 405B 0.20856

Excited State 34: 2.549-A 3.1447 eV 394.27 nm f=0.0075 <S**2>=1.374

384A -> 399A -0.14654

384A -> 400A 0.16660

385A -> 399A -0.15622

385A -> 400A 0.14870

387A -> 399A -0.35733

387A -> 400A 0.19205

388A -> 399A 0.18426

381B -> 399B 0.11951

384B -> 399B -0.29580

385B -> 399B 0.37912

387B -> 399B 0.49337

388B -> 399B 0.17835

392B -> 398B 0.11631

393B -> 398B -0.19115

Excited State 35: 2.490-A 3.1513 eV 393.44 nm f=0.0007 <S**2>=1.299

385B -> 399B 0.10113

387B -> 399B 0.12087

392B -> 398B -0.44522

393B -> 398B 0.81959

393B -> 399B 0.10821

393B -> 400B -0.11138

Excited State 36: 2.444-A 3.1850 eV 389.27 nm f=0.0069 <S**2>=1.243

390A -> 399A 0.23339

397A -> 401A 0.37258

397A -> 404A 0.14477

398A -> 405A 0.11246

398A -> 406A 0.14453

372B -> 398B -0.15539

375B -> 398B -0.14990

376B -> 398B 0.15443

382B -> 398B 0.43628

382B -> 400B 0.10349

383B -> 398B 0.25649

392B -> 399B 0.13796

393B -> 398B -0.12850

397B -> 401B 0.20832

397B -> 403B -0.10358

397B -> 405B 0.15845

Excited State 37: 2.783-A 3.2014 eV 387.28 nm f=0.0264 <S**2>=1.686

382A -> 400A 0.11193

384A -> 400A 0.16226

385A -> 400A 0.17590

387A -> 400A 0.45276

388A -> 400A -0.26939

390A -> 400A -0.13570

397A -> 404A -0.17136

397A -> 405A -0.11063

398A -> 406A -0.23925

376B -> 398B -0.10147

379B -> 398B 0.12323

386B -> 398B 0.12023

387B -> 398B 0.23882

387B -> 399B -0.19949

388B -> 398B -0.17254

389B -> 398B 0.15104

392B -> 399B 0.13362

392B -> 400B 0.12829

393B -> 399B 0.12022

397B -> 404B 0.20270

Excited State 38: 2.578-A 3.2069 eV 386.62 nm f=0.0103 <S**2>=1.411

381A -> 400A 0.12650

384A -> 400A 0.21062

385A -> 400A 0.17984

387A -> 400A 0.45815

388A -> 400A -0.13228

390A -> 400A 0.13680

397A -> 401A 0.14803

397A -> 405A 0.10665

398A -> 406A 0.31563

398A -> 407A 0.12816

385B -> 399B -0.11896

386B -> 398B -0.12700

387B -> 399B -0.18066

388B -> 398B 0.23702

389B -> 398B -0.16905

392B -> 399B -0.20104

397B -> 401B 0.28117

397B -> 404B -0.10600

Excited State 39: 2.482-A 3.2158 eV 385.54 nm f=0.0608 <S**2>=1.290

384A -> 400A -0.11038

387A -> 400A -0.14422

397A -> 401A 0.19479

397A -> 404A -0.18213

397A -> 406A 0.15989

398A -> 405A 0.23559

398A -> 406A 0.51636

398A -> 407A 0.22561

398A -> 412A -0.10228

376B -> 398B -0.12587

379B -> 398B 0.12247

382B -> 398B -0.18206

388B -> 398B -0.14285

389B -> 398B 0.11031

394B -> 398B -0.10592

394B -> 400B -0.27332

397B -> 401B 0.14380

397B -> 404B 0.17983

Excited State 40: 2.262-A 3.2215 eV 384.86 nm f=0.0442 <S**2>=1.029

390A -> 399A -0.15066

397A -> 401A 0.41228

397A -> 406A -0.16351

398A -> 401A -0.11206

398A -> 404A 0.10534

398A -> 405A -0.22586

398A -> 406A -0.41094

398A -> 407A -0.17005

394B -> 398B -0.14096

394B -> 400B -0.40267

397B -> 401B 0.44093

397B -> 405B -0.12579

Excited State 41: 2.747-A 3.2390 eV 382.78 nm f=0.0181 <S**2>=1.636

376A -> 399A -0.11043

384A -> 399A 0.16750

384A -> 400A -0.13864

386A -> 399A 0.12908

388A -> 399A 0.16539

391A -> 399A -0.12391

393A -> 399A -0.13129

394A -> 400A -0.15798

397A -> 401A 0.23218

397A -> 405A -0.16251

374B -> 398B 0.10238

385B -> 398B -0.10520

392B -> 399B -0.13293

394B -> 398B 0.17701

394B -> 400B 0.58136

397B -> 401B 0.20063

397B -> 404B 0.17847

Excited State 42: 3.014-A 3.2425 eV 382.37 nm f=0.0002 <S**2>=2.020

376A -> 399A 0.28325

377A -> 399A 0.10848

384A -> 399A -0.14112

385A -> 399A 0.14118

386A -> 399A -0.10385

387A -> 400A -0.11924

388A -> 399A -0.10649

390A -> 399A -0.12464

391A -> 399A 0.11860

393A -> 399A 0.32113

394A -> 400A -0.10643

397A -> 401A 0.18350

397A -> 402A 0.12654

374B -> 398B -0.19950

374B -> 399B -0.18705

384B -> 398B 0.21062

384B -> 399B 0.13940

385B -> 399B 0.12174

386B -> 399B -0.10626

388B -> 399B 0.13090

390B -> 398B 0.11054

390B -> 399B 0.11442

394B -> 398B 0.10915

394B -> 400B 0.39021

397B -> 401B 0.20526

Excited State 43: 3.238-A 3.2522 eV 381.23 nm f=0.0029 <S**2>=2.372

390A -> 400A 0.14752

393A -> 399A 0.47158

397A -> 405A -0.29081

397A -> 406A 0.13504

397A -> 407A -0.11596

398A -> 405A 0.10734

398A -> 406A -0.12268

374B -> 398B -0.10433

388B -> 398B 0.14332

388B -> 399B -0.12069

389B -> 398B -0.11238

392B -> 399B -0.13678

392B -> 400B -0.14457

394B -> 400B -0.20774

397B -> 402B -0.13238

397B -> 404B 0.29251

397B -> 405B 0.24846

Excited State 44: 3.120-A 3.2657 eV 379.66 nm f=0.0000 <S**2>=2.183

376A -> 399A -0.36878

393A -> 399A 0.67694

397A -> 405A 0.14876

374B -> 398B 0.30956

374B -> 399B 0.21068

374B -> 400B -0.13205

378B -> 398B -0.12368

378B -> 399B -0.10010

397B -> 404B -0.16632

Excited State 45: 2.823-A 3.2825 eV 377.71 nm f=0.0040 <S**2>=1.742

376A -> 399A 0.18964

384A -> 399A 0.11620

393A -> 399A 0.32707

397A -> 407A 0.12979

374B -> 398B -0.15811

384B -> 398B -0.11778

384B -> 399B -0.13131

387B -> 398B 0.10620

388B -> 398B -0.11359

390B -> 398B -0.20567

391B -> 398B 0.64397

391B -> 399B 0.15376

393B -> 399B -0.18392

397B -> 407B -0.10289

**ZnPc-2· B**

Excited State 1: 3.466-A 1.0803 eV 1147.64 nm f=0.0004 <S**2>=2.753

397A -> 399A 0.70378

397B -> 398B 0.18760

397B -> 399B 0.68870

397A <- 399A 0.12208

397B <- 399B 0.11803

This state for optimization and/or second-order correction.

Total Energy, E(TD-HF/TD-DFT) = -6495.78316458

Copying the excited state density for this state as the 1-particle RhoCI density.

Excited State 2: 3.277-A 1.1297 eV 1097.50 nm f=0.0024 <S**2>=2.434

397A -> 400A 0.61950

397B -> 398B 0.66995

397B -> 399B -0.18001

397B -> 400B -0.36669

397A <- 400A 0.11416

Excited State 3: 2.099-A 1.3827 eV 896.65 nm f=0.0018 <S**2>=0.852

397A -> 400A 0.16826

398A -> 399A 0.92729

398A -> 401A -0.14516

397B -> 398B -0.21123

397B -> 400B -0.18084

Excited State 4: 2.322-A 1.5975 eV 776.11 nm f=0.0307 <S**2>=1.098

397A -> 400A -0.37343

398A -> 399A 0.31331

398A -> 400A 0.11609

397B -> 398B 0.63392

397B -> 400B 0.56119

Excited State 5: 2.055-A 1.6618 eV 746.10 nm f=0.0126 <S**2>=0.806

397A -> 399A -0.12422

398A -> 400A 0.96651

397B -> 399B 0.14827

Excited State 6: 2.021-A 1.9787 eV 626.59 nm f=0.7075 <S**2>=0.771

397A -> 399A 0.67881

398A -> 400A 0.19543

397B -> 399B -0.66875

Excited State 7: 2.031-A 2.0728 eV 598.16 nm f=0.3910 <S**2>=0.782

397A -> 400A 0.64394

387B -> 399B -0.10697

397B -> 398B -0.20668

397B -> 400B 0.68726

Excited State 8: 3.465-A 2.3625 eV 524.79 nm f=0.0002 <S**2>=2.751

394A -> 399A -0.11578

395A -> 399A -0.15491

396A -> 399A 0.68363

396A -> 401A 0.10278

395B -> 399B 0.15551

396B -> 398B -0.16756

396B -> 399B -0.58985

396B -> 401B -0.10917

Excited State 9: 2.066-A 2.4208 eV 512.17 nm f=0.0078 <S**2>=0.817

398A -> 399A 0.12976

398A -> 401A 0.77878

398A -> 403A 0.17393

398A -> 404A -0.13060

393B -> 398B 0.45471

393B -> 400B 0.20165

Excited State 10: 3.302-A 2.4728 eV 501.40 nm f=0.0005 <S**2>=2.476

394A -> 400A -0.26116

395A -> 400A -0.38650

396A -> 400A -0.30839

394B -> 398B 0.18374

394B -> 400B -0.13921

395B -> 398B 0.48374

395B -> 400B -0.29987

396B -> 398B 0.38133

396B -> 400B -0.23222

Excited State 11: 3.310-A 2.5042 eV 495.10 nm f=0.0001 <S**2>=2.489

394A -> 400A -0.46849

395A -> 400A 0.30241

394B -> 398B 0.56124

394B -> 399B -0.14914

394B -> 400B -0.37022

395B -> 398B -0.31388

395B -> 400B 0.17606

Excited State 12: 3.039-A 2.6310 eV 471.24 nm f=0.0064 <S**2>=2.058

394A -> 399A -0.19810

394A -> 400A 0.19769

395A -> 400A 0.21381

396A -> 399A 0.21536

396A -> 400A -0.26349

394B -> 398B -0.10362

394B -> 399B 0.19144

395B -> 398B -0.30792

395B -> 400B 0.16273

396B -> 398B 0.68558

396B -> 400B -0.26378

Excited State 13: 3.359-A 2.6463 eV 468.52 nm f=0.0018 <S**2>=2.571

394A -> 399A 0.59324

395A -> 399A -0.28452

396A -> 400A -0.13508

394B -> 398B -0.35969

394B -> 399B -0.52487

395B -> 398B -0.14423

395B -> 399B 0.17595

Excited State 14: 3.411-A 2.6690 eV 464.54 nm f=0.0004 <S**2>=2.659

394A -> 399A 0.23282

395A -> 399A 0.64179

396A -> 399A 0.23566

394B -> 398B -0.18869

394B -> 399B -0.12288

395B -> 399B -0.59658

396B -> 399B -0.10914

Excited State 15: 2.179-A 2.6815 eV 462.38 nm f=0.0022 <S**2>=0.937

396A -> 399A 0.60223

396A -> 400A 0.17184

396B -> 399B 0.73837

Excited State 16: 2.062-A 2.6843 eV 461.89 nm f=0.0021 <S**2>=0.813

371B -> 398B 0.10007

374B -> 398B 0.14776

375B -> 398B -0.13986

376B -> 398B 0.65407

376B -> 400B 0.36886

377B -> 398B 0.41683

377B -> 400B 0.22869

379B -> 398B 0.10788

381B -> 398B -0.14030

393B -> 398B -0.16345

Excited State 17: 2.247-A 2.7500 eV 450.86 nm f=0.0107 <S**2>=1.013

395A -> 400A 0.11145

398A -> 401A -0.46404

398A -> 402A 0.19770

376B -> 398B 0.20361

376B -> 400B 0.10027

393B -> 398B 0.70301

393B -> 400B 0.18608

395B -> 398B 0.10059

Excited State 18: 2.282-A 2.7522 eV 450.49 nm f=0.0042 <S**2>=1.052

394A -> 399A 0.12277

395A -> 400A 0.43958

396A -> 400A 0.61256

395B -> 398B 0.44885

396B -> 398B 0.36913

396B -> 399B -0.13545

Excited State 19: 2.310-A 2.7676 eV 447.99 nm f=0.0002 <S**2>=1.083

394A -> 399A 0.21544

394A -> 400A 0.60533

395A -> 400A -0.35084

396A -> 400A 0.21846

394B -> 398B 0.53035

394B -> 399B -0.17213

395B -> 398B -0.19391

396B -> 398B 0.11626

Excited State 20: 2.388-A 2.8327 eV 437.69 nm f=0.0744 <S**2>=1.175

394A -> 399A -0.15777

394A -> 400A -0.16595

395A -> 400A -0.20976

396A -> 400A 0.25394

397A -> 401A -0.20648

398A -> 402A 0.72511

398A -> 404A 0.15831

381B -> 398B -0.13756

394B -> 398B -0.10928

394B -> 399B -0.11295

395B -> 398B -0.12061

396B -> 398B 0.10882

397B -> 401B -0.12215

Excited State 21: 2.190-A 2.8441 eV 435.93 nm f=0.0618 <S**2>=0.949

394A -> 399A 0.25754

394A -> 400A -0.30683

395A -> 399A -0.32891

395A -> 400A -0.29272

396A -> 400A 0.36015

398A -> 402A -0.20153

394B -> 398B -0.13510

394B -> 399B 0.43905

395B -> 398B -0.33695

395B -> 399B -0.25923

396B -> 398B 0.17093

Excited State 22: 2.301-A 2.8579 eV 433.83 nm f=0.0848 <S**2>=1.073

394A -> 399A 0.53908

395A -> 400A 0.18243

396A -> 400A -0.24350

397A -> 401A -0.21264

398A -> 402A 0.22642

381B -> 398B -0.10524

394B -> 398B 0.18650

394B -> 399B 0.54153

395B -> 398B 0.19148

396B -> 398B -0.11505

397B -> 401B -0.16766

Excited State 23: 2.170-A 2.8694 eV 432.09 nm f=0.2107 <S**2>=0.927

394A -> 399A 0.18631

394A -> 400A -0.12838

395A -> 399A 0.54759

395A -> 400A -0.22592

396A -> 400A 0.16816

397A -> 401A 0.10165

394B -> 399B 0.14417

395B -> 399B 0.65180

396B -> 398B 0.11042

Excited State 24: 3.226-A 2.8759 eV 431.12 nm f=0.0038 <S**2>=2.351

397A -> 401A 0.51671

397A -> 402A -0.17113

397A -> 403A -0.14330

398A -> 401A 0.10526

398A -> 402A 0.29855

393B -> 398B -0.12405

393B -> 399B 0.23261

394B -> 399B 0.12133

397B -> 401B 0.58867

Excited State 25: 3.375-A 2.8949 eV 428.29 nm f=0.0031 <S**2>=2.597

382A -> 399A 0.13807

384A -> 399A -0.22781

386A -> 399A -0.26444

387A -> 399A 0.29059

388A -> 399A 0.38216

389A -> 399A 0.15926

398A -> 402A -0.10456

384B -> 399B 0.18781

387B -> 398B 0.11016

387B -> 399B 0.41125

388B -> 399B 0.28588

389B -> 399B -0.30565

393B -> 398B 0.12672

395B -> 399B 0.18256

Excited State 26: 3.274-A 2.9543 eV 419.67 nm f=0.0036 <S**2>=2.429

382A -> 400A -0.13825

384A -> 400A 0.22240

386A -> 400A 0.24953

387A -> 400A -0.26780

388A -> 400A -0.32084

389A -> 400A -0.12769

398A -> 402A 0.16770

384B -> 398B -0.10480

387B -> 398B -0.32273

387B -> 399B 0.11596

387B -> 400B 0.23564

388B -> 398B -0.31086

388B -> 400B 0.19136

389B -> 398B 0.36506

389B -> 400B -0.20722

Excited State 27: 2.314-A 3.0264 eV 409.67 nm f=0.0068 <S**2>=1.089

384A -> 400A -0.10184

394A -> 400A -0.13759

395A -> 400A -0.15913

396A -> 400A -0.15829

384B -> 398B 0.10653

388B -> 398B -0.12546

389B -> 398B 0.20465

394B -> 400B 0.25923

395B -> 398B 0.13908

395B -> 400B 0.38967

396B -> 398B 0.25439

396B -> 400B 0.66272

Excited State 28: 2.948-A 3.0359 eV 408.39 nm f=0.0052 <S**2>=1.923

382A -> 399A 0.10206

382A -> 400A 0.12600

384A -> 399A -0.12042

384A -> 400A -0.15195

386A -> 400A -0.10501

388A -> 399A -0.13689

382B -> 398B -0.22149

382B -> 400B 0.11078

384B -> 398B 0.33078

384B -> 400B -0.17210

387B -> 398B 0.29219

387B -> 400B -0.16765

388B -> 398B -0.24391

389B -> 398B 0.42275

389B -> 399B 0.10573

389B -> 400B -0.17356

390B -> 398B -0.12828

394B -> 400B -0.21362

395B -> 400B -0.14449

396B -> 398B -0.10185

396B -> 400B -0.22578

Excited State 29: 3.001-A 3.0486 eV 406.69 nm f=0.0023 <S**2>=2.001

382A -> 399A -0.11665

384A -> 399A 0.14039

388A -> 399A 0.20708

389A -> 399A 0.11056

391A -> 399A 0.22604

392A -> 399A 0.12565

394A -> 400A -0.11881

397A -> 401A -0.10065

384B -> 399B -0.12174

387B -> 399B -0.11867

389B -> 398B 0.11499

389B -> 399B -0.14743

393B -> 399B 0.51900

394B -> 398B 0.14628

394B -> 400B 0.35066

395B -> 398B -0.12750

395B -> 400B -0.29967

397B -> 401B -0.12689

Excited State 30: 2.700-A 3.0491 eV 406.63 nm f=0.0009 <S**2>=1.573

382A -> 399A 0.15519

384A -> 399A -0.16660

388A -> 399A -0.26107

389A -> 399A -0.14854

391A -> 399A -0.13374

394A -> 400A -0.15690

395A -> 400A 0.14390

384B -> 399B 0.14701

388B -> 399B -0.13493

389B -> 399B 0.20122

393B -> 399B -0.21316

394B -> 398B 0.23397

394B -> 399B -0.11553

394B -> 400B 0.58651

395B -> 398B -0.14826

395B -> 400B -0.36378

Excited State 31: 3.270-A 3.0633 eV 404.74 nm f=0.0004 <S**2>=2.423

382A -> 399A 0.23217

384A -> 399A -0.23491

386A -> 399A -0.12177

388A -> 399A -0.28155

389A -> 399A -0.20459

390A -> 399A 0.10635

391A -> 399A 0.10856

392A -> 399A 0.11644

382B -> 399B -0.14414

384B -> 399B 0.21164

387B -> 399B 0.21857

389B -> 399B 0.20551

393B -> 399B 0.53010

394B -> 400B -0.14783

395B -> 400B 0.14294

397B -> 401B -0.13393

Excited State 32: 2.238-A 3.1020 eV 399.69 nm f=0.1070 <S**2>=1.003

394A -> 400A -0.14132

395A -> 400A -0.16744

396A -> 400A 0.11553

397A -> 401A 0.10339

394B -> 398B 0.11235

394B -> 400B 0.34216

395B -> 398B 0.19233

395B -> 400B 0.59247

396B -> 398B -0.18296

396B -> 400B -0.55695

Excited State 33: 3.170-A 3.1182 eV 397.61 nm f=0.0298 <S**2>=2.262

391A -> 399A -0.16092

397A -> 402A 0.51979

397A -> 403A -0.23330

397A -> 404A -0.15730

398A -> 402A 0.15376

376B -> 398B 0.12621

377B -> 398B -0.16653

381B -> 398B 0.19336

381B -> 399B -0.15028

393B -> 398B -0.11222

393B -> 400B -0.12910

394B -> 400B -0.16316

397B -> 402B 0.34900

397B -> 403B -0.25277

Excited State 34: 2.071-A 3.1416 eV 394.65 nm f=0.0038 <S**2>=0.822

382A -> 399A -0.20368

384A -> 399A 0.19651

388A -> 399A 0.44973

389A -> 399A 0.26335

382B -> 399B -0.19919

384B -> 399B 0.28029

387B -> 399B 0.16449

388B -> 399B -0.33482

389B -> 399B 0.52385

390B -> 399B -0.14982

392B -> 399B -0.10912

Excited State 35: 2.600-A 3.1792 eV 389.98 nm f=0.0075 <S**2>=1.441

392A -> 399A -0.13368

397A -> 401A -0.40226

397A -> 403A 0.17507

397A -> 404A 0.22389

372B -> 398B 0.11842

376B -> 398B 0.20000

377B -> 398B -0.20532

377B -> 400B -0.10491

381B -> 398B 0.42918

397B -> 401B 0.32007

397B -> 403B 0.30216

Excited State 36: 2.569-A 3.1992 eV 387.55 nm f=0.0417 <S**2>=1.400

382A -> 400A -0.24457

384A -> 400A 0.22640

388A -> 400A 0.59912

389A -> 400A 0.35386

390A -> 400A -0.12082

391A -> 400A 0.10319

393A -> 400A -0.10442

397A -> 401A -0.27601

397A -> 403A -0.12310

397A -> 404A -0.12147

388B -> 398B -0.13609

389B -> 398B 0.17271

397B -> 401B 0.24721

397B -> 403B -0.19047

Excited State 37: 2.530-A 3.2100 eV 386.25 nm f=0.0204 <S**2>=1.351

382A -> 400A -0.19284

384A -> 400A 0.18202

388A -> 400A 0.33812

389A -> 400A 0.19179

391A -> 399A -0.12797

392A -> 399A -0.13714

397A -> 401A 0.50211

397A -> 403A 0.15916

397A -> 404A 0.14411

389B -> 398B 0.11767

397B -> 401B -0.43947

397B -> 403B 0.25144

Excited State 38: 3.132-A 3.2316 eV 383.66 nm f=0.0034 <S**2>=2.203

378A -> 400A 0.16265

391A -> 399A -0.10358

392A -> 400A -0.22735

397A -> 402A -0.20996

397A -> 403A -0.11735

397A -> 404A -0.20174

377B -> 398B 0.12705

381B -> 398B 0.19018

381B -> 399B -0.10032

389B -> 398B 0.13247

391B -> 398B 0.41401

391B -> 399B -0.12376

391B -> 400B -0.10220

392B -> 398B 0.22480

393B -> 399B 0.23229

393B -> 400B 0.23905

397B -> 401B -0.14412

397B -> 403B -0.23961

Excited State 39: 3.311-A 3.2746 eV 378.62 nm f=0.0010 <S**2>=2.491

376A -> 399A -0.16252

381A -> 399A -0.22714

382A -> 399A 0.11410

383A -> 399A -0.24275

392A -> 399A -0.14772

397A -> 403A -0.27626

397A -> 404A 0.16868

397A -> 407A 0.17612

398A -> 405A 0.18606

374B -> 399B 0.13691

380B -> 399B 0.19851

381B -> 398B 0.12297

383B -> 398B 0.12187

383B -> 399B 0.25590

391B -> 398B 0.17275

391B -> 399B 0.11555

397B -> 401B -0.22639

397B -> 403B -0.10827

397B -> 404B 0.34526

397B -> 407B -0.11731

Excited State 40: 2.216-A 3.2767 eV 378.38 nm f=0.0026 <S**2>=0.977

397A -> 402A -0.10876

398A -> 404A 0.25082

398A -> 405A 0.89094

Excited State 41: 2.857-A 3.2832 eV 377.64 nm f=0.0294 <S**2>=1.790

391A -> 399A 0.20745

397A -> 402A 0.37794

397A -> 403A 0.10771

397A -> 404A 0.12561

397A -> 406A 0.14587

398A -> 409A -0.17645

376B -> 398B -0.18945

377B -> 398B 0.23810

381B -> 398B -0.22585

381B -> 400B -0.10997

389B -> 398B 0.11382

391B -> 398B 0.42511

392B -> 398B 0.14929

393B -> 400B 0.10699

397B -> 401B 0.13946

397B -> 402B 0.18347

397B -> 403B 0.18176

397B -> 406B 0.15447

Excited State 42: 3.161-A 3.3147 eV 374.04 nm f=0.0073 <S**2>=2.248

376A -> 399A -0.18576

391A -> 400A -0.16952

392A -> 399A -0.18963

397A -> 402A 0.10858

374B -> 398B 0.12516

374B -> 399B 0.13951

381B -> 400B 0.14380

391B -> 398B -0.17203

392B -> 398B -0.17095

393B -> 398B -0.24523

393B -> 400B 0.68452

Excited State 43: 3.399-A 3.3274 eV 372.62 nm f=0.0001 <S**2>=2.638

374A -> 399A 0.13522

376A -> 399A 0.57848

397A -> 403A -0.15920

397A -> 407A 0.12292

374B -> 398B -0.24316

374B -> 399B -0.46795

374B -> 400B 0.11449

376B -> 399B 0.15382

391B -> 398B -0.12151

393B -> 400B 0.17666

397B -> 404B 0.21066

Excited State 44: 3.076-A 3.3502 eV 370.08 nm f=0.0119 <S**2>=2.116

375A -> 399A -0.12884

376A -> 399A 0.10135

381A -> 400A -0.12002

383A -> 400A -0.11905

397A -> 404A -0.10095

397A -> 406A 0.26673

397A -> 407A -0.14964

398A -> 403A 0.20358

398A -> 404A -0.12997

398A -> 405A 0.12553

398A -> 407A 0.12138

398A -> 409A 0.16310

374B -> 398B 0.11208

377B -> 398B -0.12545

380B -> 398B 0.13203

380B -> 400B -0.10048

381B -> 398B 0.12277

381B -> 399B 0.21856

383B -> 398B 0.20408

391B -> 398B 0.27623

392B -> 398B -0.16092

397B -> 404B -0.17267

397B -> 406B 0.29534

397B -> 407B 0.12127

Excited State 45: 2.833-A 3.3644 eV 368.52 nm f=0.0003 <S**2>=1.757

375A -> 399A -0.10904

391A -> 399A 0.11121

397A -> 402A 0.12355

397A -> 407A -0.17804

398A -> 403A 0.14926

398A -> 404A -0.13771

374B -> 398B -0.10255

381B -> 399B 0.22775

391B -> 398B -0.23844

392B -> 398B 0.65547

392B -> 399B 0.11995

392B -> 400B -0.11231

397B -> 404B -0.15235

397B -> 407B 0.14351

# References

# N. Chrysochos, C. P. Constantinides, G. M. Leitus, A. Kourtellaris, D. B. Lawson, M. Deumal, J. Ribas-Ariño, M. A. Carvajal, G. A. Zissimou, C. Nicolaides, T. Trypiniotis, P. A. Koutentis, *Cryst. Growth Des.* 2023, *23*, 8939–8952.

# F. J. Céspedes-Guirao, K. Ohkubo, S. Fukuzumi, F. Fernández-Lázaro, Á. Sastre-Santos, *Chem. – Asian J.* 2011, *6*, 3110–3121.

# S. Makarov, C. Litwinski, E. A. Ermilov, O. Suvorova, B. Röder, D. Wöhrle, *Chem. – Eur. J.* 2006, *12*, 1468–1474.

# CrysAlis CCD and CrysAlis RED; Oxford Diffraction Ltd.: Abingdon, Oxford, England, 2008; Version 1.171.32.15.

# O. V. Dolomanov, L. J. Bourhis, R. J. Gildea, J. A. K. Howard, H. Puschmann, *OLEX2*: a complete structure solution, refinement and analysis program. *J. Appl. Crystallogr.* 2009, *42*, 339–341.

# L. J. Bourhis, O. V. Dolomanov, R. J. Gildea, J. A. K. Howard, H. Puschmann, The anatomy of a comprehensive constrained, restrained refinement program for the modern computing environment - Olex2 dissected. *Acta Crystallogr., Sect. A:Found. Adv.* 2015, *71*, 59–75.

# G. M. Sheldrick, Crystal Structure Refinement with SHELXL. *Acta Crystallogr., Sect. C: Struct. Chem.* 2015, *71*, 3–8.

# P. R. Edgington, P. McCabe, C. F. Macrae, E. Pidcock, G. P. Shields, R. Taylor, M. Towler, J. Van De Streek, *Mercury*: visualization and analysis of crystal structures. *J. Appl. Crystallogr.* 2006, *39*, 453–457.

# J. Krumsieck, M. Bröring, *PorphyStruct* – A Digital Tool for the Quantitative Assignment of Non-Planar Distortion Modes in Four-Membered Porphyrinoids. *Chem. - Eur J.*, 2021, *27*, 11580-11588.

# Gaussian 16, Revision C.01; Frisch, M. J.; Trucks, G. W.; Schlegel, H. B.; Scuseria, G. E.; Robb, M. A.; Cheeseman, J. R.; Scalmani, G.; Barone, V.; Petersson, G. A.; Nakatsuji, H.; Li, X.; Caricato, M.; Marenich, A. V.; Bloino, J.; Janesko, B. G.; Gomperts, R.; Mennucci, B.; Hratchian, H. P.; Ortiz, J. V.; Izmaylov, A. F.; Sonnenberg, J. L.; Williams-Young, D.; Ding, F.; Lipparini, F.; Egidi, F.; Goings, J.; Peng, B.; Petrone, A.; Henderson, T.; Ranasinghe, D.; Zakrzewski, V. G.; Gao, J.; Rega, N.; Zheng, G.; Liang, W.; Hada, M.; Ehara, M.; Toyota, K.; Fukuda, R.; Hasegawa, J.; Ishida, M.; Nakajima, T.; Honda, Y.; Kitao, O.; Nakai, H.; Vreven, T.; Throssell, K.; Montgomery, J. A., Jr.; Peralta, J. E.; Ogliaro, F.; Bearpark, M. J.; Heyd, J. J.; Brothers, E. N.; Kudin, K. N.; Staroverov, V. N.; Keith, T. A.; Kobayashi, R.; Normand, J.; Raghavachari, K.; Rendell, A. P.; Burant, J. C.; Iyengar, S. S.; Tomasi, J.; Cossi, M.; Millam, J. M.; Klene, M.; Adamo, C.; Cammi, R.; Ochterski, J. W.; Martin, R. L.; Morokuma, K.; Farkas, O.; Foresman, J. B.; Fox, D. J. Gaussian, Inc., Wallingford CT, 2016.

# A. D. Becke, Density-functional thermochemistry. III. The role of exact exchange. *J. Chem. Phys*. 1993, *98*, 5648–5652.

# Gaussian Technical Note ‘Creating UV/Visible Plots from the Results of Excited States Calculations’, <https://gaussian.com/uvvisplot/>. Accessed 10-10-2025.
